# Evolutionary transitions toward pair living in nonhuman primates as stepping stones toward more complex societies

**DOI:** 10.1126/sciadv.aay1276

**Published:** 2019-12-18

**Authors:** Peter M. Kappeler, Luca Pozzi

**Affiliations:** 1 Behavioral Ecology and Sociobiology Unit, German Primate Center–Leibniz Institute for Primate Research, Göttingen, Germany.; 2 Department of Sociobiology/Anthropology, University of Göttingen, Göttingen, Germany.; 3 Department of Anthropology, The University of Texas at San Antonio, San Antonio, TX, USA.

## Abstract

Nonhuman primate societies vary tremendously in size and composition, but how and why evolutionary transitions among different states occurred remains highly controversial. In particular, how many times pair living evolved and the social states of the ancestors of pair- and group-living species remains contentious. We examined evolutionary transitions in primate social evolution by using new, independent categorizations of sociality and different phylogenetic hypotheses with a vastly expanded dataset. Using Bayesian phylogenetic comparative methods, we consistently found the strongest support for a model that invokes frequent transitions between solitary ancestors and pair-living descendants, with the latter giving rise to group-living species. This result was robust to systematic variation in social classification, sample size, and phylogeny. Our analyses therefore indicate that pair living was a stepping stone in the evolution of structurally more complex primate societies, a result that bolsters the role of kin selection in social evolution.

## Teaser

A new scenario for primate social evolution: Pair living evolved repeatedly from solitary ancestors.

## INTRODUCTION

The comparative study of animal societies has revealed spectacular interspecific diversity in the size, composition, and cohesion of social units (*1*). Because the evolution of group living has been suggested to represent one of the major transitions in evolution, it continues to attract much scrutiny. While the key ultimate costs and benefits of group living have been identified, and the principles of collective behavior have been unraveled, how different species-specific social systems evolved with relation to species’ phylogenetic histories is still subject to much ongoing research and controversy (*2*–*9*). Consideration of phylogenetic relationships in modeling interspecific variation in behavioral phenotypes is indicated because phylogenetic signals have indeed been detected in social traits at the species level, despite social behavior perceived as being more labile or flexible in response to ecology than other traits.

Because primates are relatively large-bodied, terrestrial, mostly diurnal, and because they gave rise to the human lineage, their social systems have been studied in more detail than those of other mammals. Their social organization, defined as the size, composition, and cohesion of social units (*10*), varies widely across species: About one-third of all extant primate species are solitary, about one-fifth are pair living, and the rest live in multimale, multifemale (MMMF) groups, some of which include hundreds of individuals that are organized hierarchically across multiple levels. Yet, how and why different levels of social complexity evolved, i.e., how many evolutionary transitions among which types of social organization occurred and which selective factors promoted the possible state transitions, remains controversial.

Pair living plays a key role in this controversy. It refers to a social organization in which one adult male and female live together and coordinate their activities (*10*). Pair living is sometimes used synonymously with monogamy, but the latter refers to the mating system, which can vary widely within this type of social organization because of variable levels of extra-pair matings; these two components of a social system should therefore be separated conceptually (*11*). The evolution of pair living is not only of fundamental interest because this type of social organization can be found among many contemporary and historic human populations (*12*, *13*) but also because it represents a theoretical puzzle in social evolution in most taxa, including primates (*5*, *14*). Specifically, since males of most animal species have a much higher potential for producing offspring per unit time than females, evolutionary biologists have struggled to identify selective advantages that would more than compensate for the loss of potential reproduction suffered by males that limit their reproductive activities to a single female. In mammals, this problem is exacerbated because internal gestation and subsequent lactation are essential aspects of maternal care that markedly reduce the rate at which females can produce offspring compared to males. Thus, because male mammals that bond with a single female presumably forego additional mating opportunities, and because, in contrast to many birds, rates of extra-pair paternity among monogamous mammals are generally low, pair living is part of a male reproductive strategy requiring explanation (*15*, *16*).

Of several explanations for the evolution of mammalian pair living put forward, only two have enjoyed repeated empirical support: The female spacing and the paternal care hypotheses. The female spacing hypothesis posits that females pursue reproductive strategies that are not limited by the number of mates but by access to resources (*5*). Thus, characteristics of their diet and other ecological factors determine the distribution of females in space. Under certain ecological conditions, females experience intense feeding competition and space out widely in response, limiting males’ ability to monopolize access to multiple females. In support of this hypothesis, comparative studies across all mammals demonstrated that extant pair-living species occur at lower densities and with less range overlap with neighbors than females in solitary species (*5*). However, one comparative study failed to find support for this hypothesis (*17*).

The paternal care hypothesis is theoretically founded on two potential types of male care (*6*). First, females in some species may depend on obligate paternal care in the form of protecting, warming, carrying, or provisioning to successfully rear offspring. Males bonded to a single female may nonetheless enjoy greater reproductive success than males pursuing alternative reproductive strategies by investing paternal care into offspring they have sired with great probability (*18*). Second, paternal care may take on the form of establishing protective associations with a mother and her infant(s), allowing males to reduce the risk of infanticide by strange males for their putative offspring. The evidence in favor of this hypothesis is currently mixed.

Studies of some species excluded infanticide risk as a potential determinant of pair living because infanticide has never been observed, but the absence of infanticide may also indicate that male protection is particularly effective. Recent comparative studies revealed that male infanticide was least prevalent among pair-living species (*19*) but that only the presence of infanticide reliably increased the shift to pair living among primates (*6*). Thus, infanticide risk was an important selective agent in primate social evolution, but it remains highly controversial whether reduction of infanticide risk was a driver or a consequence of evolutionary transitions to pair living (*20*).

Despite the apparently contradictory evidence for these two hypotheses, they may not be mutually exclusive. One of the first reviews of mammalian monogamy already suggested that spacing is the key for facultatively monogamous species and paternal care for obligately monogamous ones (*21*), but this idea remains to be formally tested. Thus, if one factor was instrumental in promoting the origin of pair living and monogamy, and the other became secondarily advantageous, for example, through new opportunities for paternal care after changes in female spacing or infanticide risk, then these two hypotheses could be reconciled. This crucial distinction between the origin and maintenance of a social system highlights the fundamental importance of the evolutionary transitions to pair living because different processes and selective factors can and must be invoked to explain transitions from either solitary or group-living ancestors (*13*, *15*, *22*).

In this study, we assessed the evolutionary transitions in the phylogenetic history of primates that led to the emergence of pair-living societies and, indeed, the other complex forms of social organizations that are discernible in primate species. We did so with a view of gaining insights into distinguishing between the two aforementioned hypotheses by elucidating their assumptions. Furthermore, the patterns of evolutionary transitions among different types of social organization also have important implications for the reconstruction of the evolution of group living and kin-based cooperation because the ancestral state for transitions to more complex bonded groups may vary (*2*–*4*, *8*, *23*). Thus, reconstructing the evolutionary transitions during primate social evolution is fundamental not only to understanding both the evolution of pair living and of structurally more complex social systems but also for offering predictions about specific selective factors either favoring transitions to another social system and/or promoting their subsequent stabilization.

However, the most recent primate studies addressing this problem diverge in the results of their phylogenetic reconstructions of the number and direction of evolutionary transitions toward pair living. A study covering all mammals revealed seven transitions to pair living among primates, six of which were from solitary and only one from group-living ancestors (*5*). Another study focusing on the social evolution of primates revealed that pair living evolved six times among primates but, in every case, from group-living ancestors (*4*). These two studies are also in disagreement over the number of pair-living primate species. The study spanning all mammals classified 29% of the 361 primate species included in their study as pair living (*5*), whereas the study focusing on primates rated 19% of 230 taxa as pair living (*4*). Furthermore, both studies used not only different criteria for counting transitions but also different operational definitions of pair living, which, however, was labeled as (social) monogamy in both studies. The study of Lukas and Clutton-Brock (*5*) classified the breeding status of female, whereas the study of Shultz *et al.* (*4*) used more general intersexual association patterns, indicating that a lack of a general consensus on operational definitions can contribute to divergence among the outcome of otherwise very similar analyses (*11*, *24*).

Because a recent comparative analysis of brain size variation among primates (*25*) also highlighted the effects of classification bias on the outcome of comparative analyses, we chose to classify species used in the present analyses anew based on a new, independent data source and to use several classification schemes with different numbers of categories to systematically explore the consequences of classification biases in comparative studies. Our dataset is also substantially larger than that used in the study of Shultz *et al.* (*4*) and uses different phylogenies to systematically explore the effects of selecting a particular phylogeny as well.

**Fig. 1:**
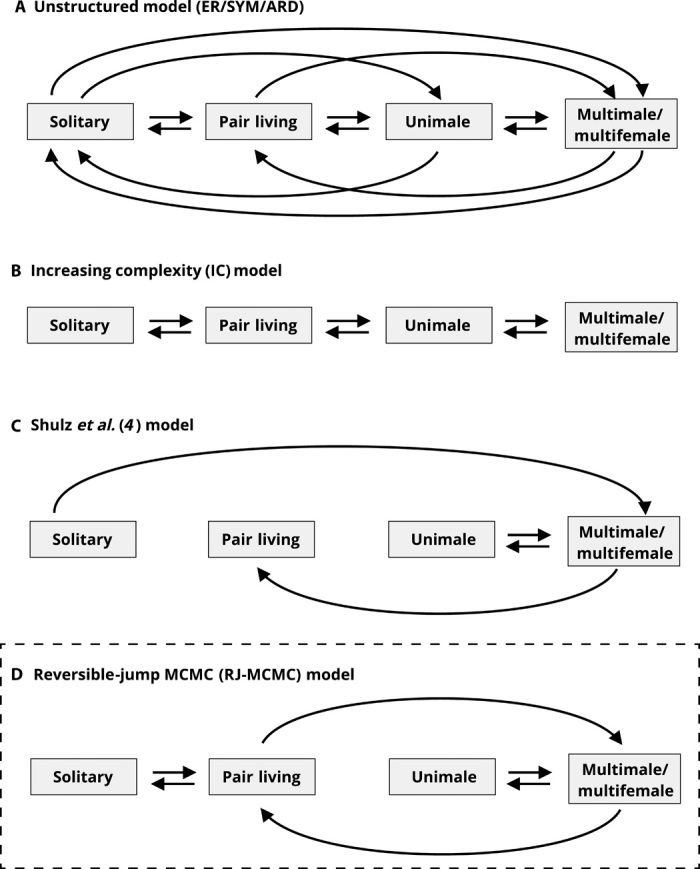
Alternative evolutionary models of social evolution. Arrows represent permitted transitions between different social organizations for each model. (**A**) Unstructured models: Under this model, all possible transitions are allowed. ER: All rates are fixed to a single optimized rate parameter; SYM Forward and reverse rates between two states are identical; ARD: Rates are fully independent. (**B**) Increasing complexity (IC) model: Transitions are only allowed between solitary and pair living, pair living and unimale groups, and unimale groups and multimale organization. (**C**) Shultz *et al.* (*4*): Transitions are allowed from solitary to multimale and from multimale to pair living and to unimale and back. (**D**) Reversible-jump–derived model (RJ-MCMC): Transitions are permitted from solitary to pair living, from pair living to multimale, and from multimale to unimale. All transitions are reversible. The RJ-MCMC (dashed box) was the preferred model using Bayes factor (see Table 1).

Thus, the aims of our study were to examine the evolution of pair living in primates in the more general context of the evolution of primate social organization to begin resolving existing discrepancies in the literature summarized above. By using a vastly expanded dataset, by permuting phylogenies and sample sizes, and by using alternative species classifications, we compare the ability of six different models (Fig. 1) to explain the evolutionary transitions in social organization. The results have important implications for distinguishing between hypotheses that invoke different factors in the origin and maintenance of pair living because transitions toward pair living from solitary or group-living ancestors rely on very different processes (*22*). Moreover, these analyses provide an essential basis for reconstructions of the evolutionary origins of structurally more complex forms of sociality, and kin selection, in particular, plays very different roles in different scenarios, depending on the inferred ancestral condition of group-living taxa. These analyses therefore inform studies of vertebrate sociality more generally.

## RESULTS

### Phylogenetic signal

To evaluate the evolution of pair living across primates, we mapped their social organization on a phylogenetic tree including 362 species (table S1). Each species was classified as solitary (S), pair living (P), or group living [the latter being either unimale (UM) or multimale (MM) in a four-state classification]. We first evaluated the level of phylogenetic signal in the dataset. Closely related taxa were found to have generally similar social systems. Using Blomberg’s *K*, the phylogenetic signal was significantly different from a chance distribution of sociality across species (three states: *K* = 0.929, *z* score from randomization = −6.057, *P* < 0.001; four states: *K* = 0.685, *z* score from randomization = −6.475, *P* < 0.001). The maximum likelihood estimate of Pagel’s lambda for the four social states (S-P-UM-MM) was 0.97 [log = −165.177, significantly different from a λ = 0; log_0_ = −458.106, *P* < 0.001; corrected Akaike Information Criterion (AICc) = 585.859]. Classifying unimale and multimale species together as “group living” led to identical results (λ = 0.98; log = −85.066, significantly different from a λ = 0; log_0_ = −36.996, *P* < 0.001; AICc = 503.860). The *D* statistics, another indicator of phylogenetic signal, varied between different traits, with values slightly lower than 0 for solitary (−0.261) and pair-living (−0.094) social systems, higher than 0 for unimale (0.139), and around 0 (0.004) for multimale, multifemale social system. When we clustered both unimale and multimale in a single category (G, group living), the *D* statistic was slightly negative (−0.270). The evolution of all traits was significantly different from random evolution, and, in all cases, it did not differ significantly from the Brownian model (*P* > 0.05; table S2).

### Model selection

**Fig. 2:**
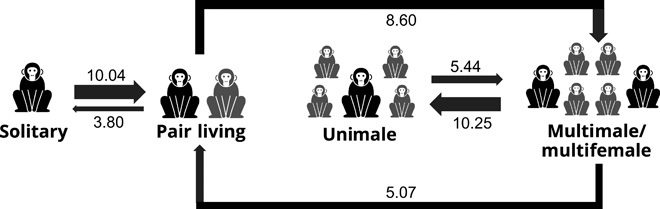
Transition scheme for RJ-MCMC Arrows with different weights representing likelihood of transition from one state to the other. Numbers indicate the average number of inferred changes between states across 10,000 mapped trees.

We evaluated six alternative models of social evolution within primates (Fig. 1). We first estimated a model directly from the data, using the reversible-jump approach implemented in BayesTraits [reversible-jump Markov chain Monte Carlo (RJ-MCMC)]. This procedure carries out an MCMC analysis in which the number of model parameters changes from one iteration to the next. The full model allowed each of the 6 rate parameters for the three-state scheme (and 12 parameters for the four-state scheme) to be estimated separately, while other models restrict the values of some rate parameters to equal the values of other rate parameters. For a four-state scheme, the results of the RJ-MCMC indicated a model in which pair living represents a stepping stone between solitary and multimale/multifemale groups (posterior support of 79.0%). Direct transitions between solitary and group living (either UM or MM) did not occur. Transitions to a unimale social organization only occurred from multimale groups. Although at different rates, all transitions that were not set to zero were considered reversible (see Fig. 2). For the three-state scheme, the RJ-MCMC model was identical to the increasing complexity (IC) model (posterior support of 78.4%), with the pair-living state being a necessary step between solitary and group living (see fig. S1).

Once we identified the model selected by the RJ-MCMC approach, we statistically compared this model against five alternative models using log Bayes Factors (BFs; see Materials and Methods). For both three- and four-state schemes, the reversible-jump–derived model was the one supported by the BF analyses (Table 1). For the four-state scheme, the RJ-MCMC model was strongly supported against the symmetrical (SYM) model (BF = 13.63), the IC model (BF = 15.05), the all-rates-different (ARD) model (BF = 23.47), and the equal rate (ER) model (BF = 25.63). The model with the least support (BF = 51.58) in our analyses was the one proposed by Shultz *et al.* (*4*), implementing frequent transitions from group to pair living. Results were largely congruent for the three-state scheme. The IC model selected by the reversible-jump approach was slightly more supported than the SYM model (BF = 2.80), more strongly supported than the ER (BF = 5.71) and the ARD (BF = 8.76) models, and very strongly supported compared to the modified version proposed by Shultz *et al.* (BF = 48.84).

**Table 1:** Comparison of alternative model performance.

**Model**	**Rank**	**Parameters**	**Marginal likelihood**	**Log_10_[BF]**	**BF interpretation**
**Four-state scheme**					
**RJ-MCMC model**	**1**	**6**	**−133.43**		
SYM model	2	6	−140.24	13.63	Very strong
IC model	3	6	−140.95	15.05	Very strong
Unconstrained model (ARD)	4	12	−145.16	23.47	Very strong
ER model	5	1	−146.24	25.63	Very strong
Shultz *et al.* (*4*) model	6	4	−159.21	51.58	Very strong
**Three-state scheme**					
**IC model**	**1**	**4**	**−79.07**		
SYM model	2	3	−80.47	2.80	Positive evidence
ER model	3	1	−81.93	5.71	Strong
Unconstrained model (ARD)	4	6	−83.45	8.76	Strong
Shultz *et al.* (*4*) model (modified)	5	2	−103.49	48.84	Very strong

We also took into account phylogenetic uncertainty by using a posterior distribution of dated phylogenies from 10kTrees (*26*). Model selection resulted in similar results. For the four-state scheme, the reversible-jump approach indicated the same model as described above, with pair living as a stepping stone between solitary and multimale/multifemale groups (posterior support of ~71%). For the three-state scheme, the IC model was strongly supported (~81%) over all the other models (see tables S3 to S6 for full results).

### Ancestral state reconstruction

We conducted ancestral state reconstruction analyses using the model selected by the reversible-jump approach. We used two different approaches to reconstruct the evolutionary history of social organization across the primate tree: the MultiState function implemented in BayesTraits 3.0 and a stochastic mapping using the “make.simmap” function of the “phytools” package in R. Ancestral states in stochastic mapping were estimated with the maximum likelihood approach using the function “ace” in the R package “ape”.

Model selection among the six alternative evolutionary models for stochastic mapping indicated a high support for the RJ-MCMC model for the four-state scheme, with weighted AIC (AICw) equal to 0.670 (AIC = 379.171). The second most likely model was the symmetrical one (SYM) with AICw equal to 0.327 (AIC = 380.601). All other models were poorly supported (AICw < 0.001). For the three-state scheme, the SYM model had the highest support (AICw = 0.661; AIC = 207.972) over the IC model (AICw = 0.274; AIC = 209.731). All other models had values of AICw lower than 0.050. The SYM model and the IC model showed similar AIC values, and they are actually very similar to each other since they both indicated zero rate of transition between the solitary state and group living. The SYM model in this case can therefore be considered a subset of the IC model where rates of transitions are symmetrical instead of being independent.

**Fig. 3:**
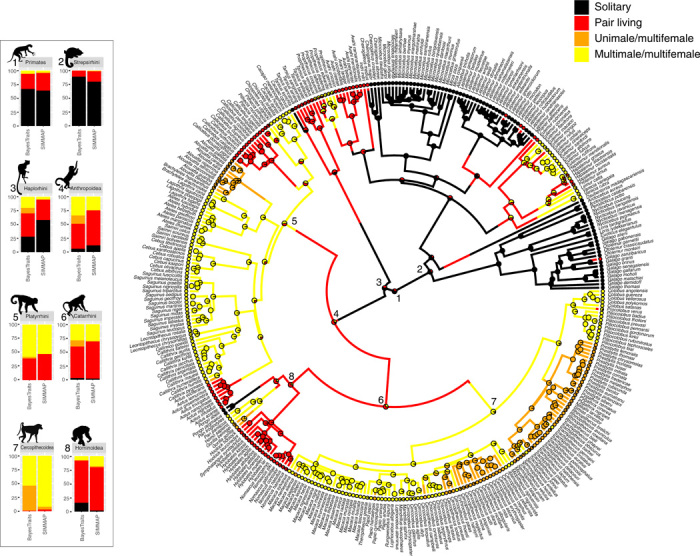
Primate phylogeny showing ancestral state reconstructions for sociality under the RJ-MCMC–derived model of evolution. Branches and tips are colored for solitary (black), pair living (red), unimale (orange), and multimale (yellow) using one tree randomly selected from the 10,000 trees in the stochastic mapping process. Pies at each node are derived from ancestral state reconstruction using the function ace (R package ape v.5.0). Ancestral state reconstruction using both stochastic mapping (SIMMAP/ace) and BayesTraits for eight main nodes (1: primate root; 2: Strepsirhini root; 3: Haplorhini root; 4: Anthropoidea root; 5: Platyrrhini root; 6: Catarrhini root; 7: Cercopithecoidea root; 8: Hominoidea root) are reported in the stacked bar graphs on the left.

Different approaches resulted in similar reconstructions of the evolutionary history of social organization across primates (Fig. 3). Both BayesTraits analyses and stochastic mapping indicated a high probability of a solitary state for the most recent common ancestor of all primates (0.64 to 0.67), the strepsirhine root (0.80 to 0.88), the lemuriform root (0.70 to 0.79), and the lorisiform root (0.99 to 1.00). Group living was reconstructed for the common ancestor of Cercopithecoidea, Cercopithecidae, and Colobinae. However, while stochastic mapping strongly inferred a multimale/multifemale organization, BayesTraits reconstruction revealed also considerable support for an ancestral unimale social organization (Fig. 3). Both analyses showed a relatively high level of ambiguous reconstruction among states for several nodes, including the root of Haplorrhini, Anthropoidea, Catarrhini, and Platyrrhini (Fig. 3). These results were consistent across both the three-state and the four-state schemes (Fig. 3 and fig. S2).

Both BayesTraits and stochastic mapping analyses specified pair living as a necessary stepping stone toward higher levels of structural social complexity (UM or MM groups). In the four-state scheme analyses, results from stochastic mapping indicated a high average number of transitions across 10,000 trees from solitary to pair living (10.036), from pair living to multimale social organization (8.599), and from multimale to unimale social organization (10.250). Reversals were overall less common (between 3.803 and 5.437; Fig. 2 and table S7). Similar results were obtained for the three-state scheme, with numerous transitions from solitary to pair living (9.375) and from pair living to group living (7.147), reinforcing the idea of IC from solitary to group living, passing through a critical intermediate phase of pair living (table S7).

### Influence of taxonomic sampling

Phylogenetic analyses can be affected not only by the topology used but also by taxon representation. Since we obtained significantly different results compared to previous analyses by Shultz *et al.* (*4*), we investigated whether some of our results might be due to higher taxon sampling. In our study, we included 362 species compared to 217 species in the previous study (*4*), representing an increase of ~67%. To explore the influence of taxonomic sampling in our dataset, we created multiple subsets using 10 sampling schemes: 344 taxa (representing 95% of the taxa represented in the original dataset), 326 taxa (90%), 308 taxa (85%), 290 taxa (80%), 272 taxa (75%), 253 taxa (70%), 235 taxa (65%), 217 taxa (60%), 199 taxa (55%), and 181 taxa (50%). For each scheme, 10 independent subsamples were randomly created and analyzed. Overall, a total of 100 datasets were run. For each of these 100 subsets, we explicitly tested the six alternative models described above (ER, SYM, ARD, Shultz, RJ-MCMC, and IC) and identified the most likely model.

Our results indicated consistent support for the RJ-MCMC model, although this support becomes less consistent as a function of taxon representation (fig. S3). When we included all 362 species, we obtained a high level of support (average AICw = 0.99), whereas support decreased to 0.76 when using only 70% of the randomly sampled taxa. We also tested the support for each of the six alternative models using only the 217 taxa included in Shultz *et al.* (*4*). Once again, the RJ-MCMC model obtained the highest support with average AICw = 0.96, followed by the SYM model with average AICw = 0.03. The latter model was the one that obtained the second highest support across all the different subsets (average AICw ranging between 0.03 and 0.17). All other models were poorly supported with AICw values less than 0.05 in any of the sampling schemes used. Our analyses therefore indicate that, although random taxonomic sampling slightly affects the support for alternative models, the overall results consistently support the scenario that pair living served as a stepping stone toward structurally more complex forms of sociality.

## DISCUSSION

Our analyses reveal several new insights about social evolution and offer explanations for the disparate results and conclusions of previous studies. In particular, our study contributed to the illumination of the evolution of primate pair living, which represents an enigma in the evolutionary biology of mammals and is widely found across human societies. In addition, we resolve a long-standing controversy about the evolution of pair living by finding support for assumptions of the female spacing hypothesis. Specifically, by systematically controlling for the social classification of species, sample size, and phylogeny, we demonstrate that, in contrast to previous analyses, pair living among primates has evolved most often from solitary ancestors and served as a stepping stone toward the evolution of structurally more complex societies. This result has implications for explaining the origins and maintenance of complex societies among primates and beyond.

First, as in several previous studies, we found evidence for strong phylogenetic signal in primate social evolution. The social organization of cercopithecines and eulemurs, for example, was found to be highly invariant, despite the great ecological diversity exhibited by this clade (*27*, *28*). Social structure, which represents an independent component of a social system, was also better explained by phylogeny than by ecology among macaques (*29*). Compared to morphological and life history traits, however, aspects of primate social organization have moderate to low phylogenetic signal (*30*). Variation in social organization is also found within primate species, across either space or time, and is best predicted by group size (*31*), but this variation is overall relatively weak. Because transitions among social states occurred independently in all major clades, they appear to reflect convergent adaptations in various environments, indicating that explicit phylogeographic analyses would be interesting to examine the conditions under which various transitions occurred. Thus, although other traits are much more strongly conserved, primate social organization can be, evolutionarily speaking, rather inert at lower taxonomic levels, suggesting that evolutionary transitions among social states of closely related species must be driven by powerful selective forces, such as fundamental changes in predation risk associated with changes in circadian activity (*4*).

Second, the present analyses clarify the patterns of evolutionary transitions toward pair living because multiple analyses converge on the identification of solitary ancestors as the most likely origin of pair living. The observed overall pattern of transitions toward pair living based on a Bayesian analytical framework resembles that described for all mammals based on previous parsimony reconstructions (*5*). The best supported stepping stone model also enjoyed support more than 75% of the times, whereas the best model in the analyses of Shultz *et al.* (*4*) was only supported by 18% of their simulations. Our systematic permutation of species classifications (three- or four-state), phylogenies, and sample sizes also confirms earlier suggestions that this discrepancy between studies is most likely due to a contrast in the classification of social systems as a result of different operationalizations of pair living and sample inflation (table S1). Our analyses therefore highlight the importance of a conceptual separation between a species’ social organization, mating system, and care system and its consequences for their operationalization (*11*, *32*).

Last, our key finding has important implications for our understanding of the factors driving evolutionary transitions toward both pair living and group living. While we did not test these predictions directly in the present analyses, the observed pattern of likely evolutionary transitions allows indirect inferences about the origins of primate pair living. Given the support we found for the prevalence of a solitary social organization among the ancestors of pair-living primates, we interpret this result as indirect support for the female spacing hypothesis, which postulates that increasing female competition and intolerance among territorial females played a key role at the origin of this transition (*5*). When females are spaced out, males associate with one female and defend her against neighboring rivals, but there are no obvious selective advantages for males promoting this transition. However, protection from infanticide risk can be invoked as a secondary force stabilizing the maintenance of pair living (*6*). In the few cases where secondary transitions from group living ancestors to pair living occurred, female competition was also the most probable driver (*33*).

**Fig. 4:**
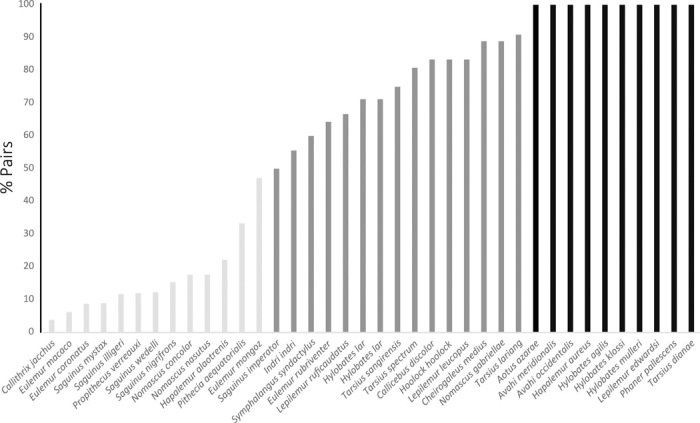
Proportion of pairs among primate social units with at least some pairs. Some species appear to be obligatorily pair living because all of their social units consists of only one pair of adults (black). In other species, more than 50%, but less than 100%, of social units consist of pairs (dark gray). The remaining species are characterized by not only a predominance of groups with three or more adults but also some pairs (light gray). Total sample size is 1081 social units from 38 species.

In considering the subsequent transitions from pairs to larger groups, two points are worth highlighting. First, in some species, pair living appears to be obligate and stable as 100% of social units consist of an adult pair. However, in many other species, a variable proportion of social units contains an additional adult member (Fig. 4), most often a second female. These facultatively pair-living species not only reflect the problems associated with operationalizing pair living in the face of intraspecific variation but they also indicate the existence of two routes toward group living. In some cases, the additional adults may represent adult or adult-sized offspring of the resident pair that have delayed natal dispersal; otherwise, they may reflect local heterogeneity in habitat quality or individual variation in male monopolization potential that permit the formation of groups with three or more adult members (*34*). Only data from long-term field studies of pair-living species can ultimately distinguish between these possibilities (*35*).

Second, sex-biased natal dispersal and the resulting kin structure are key factors in reconstructing transitions toward group living. Previous analyses suggested that sex-biased natal dispersal followed the shift toward group living rather than preceding it (*4*). In this case, female philopatry could facilitate kin selection and the emergence of cooperation only after the transition to group living. However, under both scenarios leading to the formation of occasional trios in pair-living species, it is likely that coresident adult females are close kin because, even in solitary species, daughters tend to settle close to their maternal range and young males disperse from their natal range (*36*). Studies of social insects and birds revealed that evolutionary transitions to group living were facilitated by monogamous mating and the production of related offspring that can remain in the group to receive indirect fitness benefits (*2*, *3*). This prediction is currently difficult to test in primates because the genetic mating system of only a handful of pair living species has been studied (*18*), and the distinction between singular and plural breeders (*5*) does not recognize the male contribution to offspring’s kin structure. The present analyses also make it highly unlikely that, in contrast to suggestions by previous analyses (*4*), stable groups originated from so-called “loose or unstable aggregations” because a social system with these characteristics does not exist; at least not among the lemurs for which it has been invoked (*33*). Thus, kin selection among closely related and bonded females can be implicated as a selective force at the origin of structurally complex primate societies, facilitating a range of cooperative behaviors, as invoked by the socioecological model for primate social evolution (*37*), and additional studies of the genetic mating system of pair-living primates will allow future tests of this suggested mechanism.

In conclusion, the results of this study indicate that pair living is not a highly derived social system among primates. Instead, it represents an evolutionary stepping stone between a solitary lifestyle and socially more complex systems. Female competition was presumably instrumental in the transition from a solitary social organization, and female cooperation with kin in facultatively pair-living species operated during the subsequent evolution of larger groups. Human pair bonding within larger social units therefore had other evolutionary origins than pair living among most nonhuman primates, because none of our most recent common ancestors were solitary (*13*, *15*). Opportunities for paternal care, including a reduction in infanticide risk, may have contributed to the maintenance of pair living once it evolved in primates and humans.

## MATERIALS AND METHODS

### Data collection and compilation

We compiled a dataset including 362 species of primates for which information regarding their social system was available. Our primary source was an online repository on primate socioecology (www.alltheworldsprimates.org/Home.aspx), where information on every primate species’ natural history, life history, and ecology, including its social organization, has been assembled and coded by designated experts with first-hand experience of studying a given taxon based on identical instructions (*38*). This dataset is therefore new and independent of previous studies. Our secondary source was a compilation of data on primate group size and composition in wild, diurnal, gregarious species including 3530 records on 137 species from 238 study sites collated from the primary literature by M. Stojan-Dolar (University of Ljubljana). We assigned social system categories based on two different classification schemes (see also table S8). In the first scheme (three-state), species were classified as solitary (S), pair living (P), and group living (G) using the definitions of Kappeler and van Schaik (*10*); in the second classification scheme (four-state), we followed the approach used by Shultz *et al.* (*4*), splitting groups further into species with UM and MM groups (table S1). Whenever available, we considered intraspecific variability in assigning social system categories. We ran analyses using both unique and polymorphic states in case multiple social systems have been reported for a particular species.

### Primate phylogeny

Analyses were conducted on the phylogenetic tree proposed by Springer *et al.* (*39*), which represents the most complete primate phylogeny available to date. This phylogenetic reconstruction is based on a concatenation of 69 nuclear gene segments and 10 mitochondrial gene sequences for 367 primate species. We pruned this tree down to 362 species for which data regarding the social organization were available (see “Data collection and compilation” section above). Nodes in the phylogeny were dated on the basis of relaxed clock analyses using 14 fossil-calibrated nodes and the software MCMCTree [see (*39*) for details]. To evaluate whether our results were sensitive to phylogeny, we also ran the model selection analyses (see below) using 1000 different trees from the 10kTrees Project [version 3; (*26*)], which were created using Bayesian phylogenetic methods and sampled in proportion to their probability. Although the 10kTrees approach has some important limitations (*40*), phylogenetic trees obtained from this website have been used in numerous primate comparative studies. This primate phylogeny was inferred from six mitochondrial sequences and three autosomal genes for 301 species. The nodes of the trees are dated using mean molecular branch lengths from the Bayesian analysis and six known fossil calibration points. Overall, 278 species overlapped between the 10kTrees phylogeny and our species dataset.

### Phylogenetic signal

Phylogenetic signal in data indicates whether related species are more similar to each other in social organization than expected by chance. To quantify phylogenetic signal, we calculated both Blomberg’s *K* and Pagel’s lambda using functions in the R package “picante” and the fitDiscrete function in the Geiger package, respectively. We also used the *D* statistic (*41*) as a measure of phylogenetic signal for each individual social system using the “phylo.d” function in “caper”. *D* is applicable only for binary traits, so we codified each state as either absent (0) or present (*1*). A *D* of 0 indicates that a trait evolves on a tree following the Brownian model (strong phylogenetic signal), and a *D* of 1 indicates that a trait evolves following a random model (no phylogenetic signal). If *D* is negative, then the trait evolves in a more conserved way than predicted by the Brownian model. We conducted a simulation (1000 permutations) to test whether an estimated *D* was significantly different from the predictions of a random or a Brownian style evolution.

### Model selection

The MultiState option in BayesTraits 3.0 (*42*); www.evolution.rdg.ac.uk/BayesTraitsV3/BayesTraitsV3.html) was used to identify the model best supported by the data. We initially used the reversible-jump (RJ) procedure, using different sets of priors (tables S3 to S6). Each MCMC simulation was run for 100 million iterations sampled every 1000 generations, with the first 25 million iterations discarded as the burn-in. We assumed convergence when the posterior distribution was approximately normal, and trace of harmonic mean log-likelihoods did not show large jumps across runs. Models visited by the Markov chain were ranked in order of their posterior probability. We also examined rate parameters across the Markov chain plotted in Tracer and the effective sample sizes for the parameters of interest (ESS > 200). Analyses in BayesTraits were also run using polymorphic states (two or more states recorded for a species).

We then compared alternative evolutionary models of social evolution using log BFs. We constructed six different models of social organization. First, we used an ER model, thus simulating equal likelihood for all transitions (Fig. 1A). Second, we fit a six-parameter (three-state scheme: three parameters) SYM model (Fig. 1A), where forward and reverse transitions share the same parameter. Third, rates were allowed to vary freely without constraint to produce a 12-parameter ARD model (Fig. 1A). In the case of a three-state scheme, this model estimated six parameters. The fourth model was an “IC” model where transitions were restricted so that movements were only allowed between solitary and pair living, pair living and unimale groups, and unimale groups and multimale organization (Fig. 1B). In the case of a three-state scheme, transitions were only allowed between solitary and pair living and pair living and group living. The fifth model we tested was the one proposed by Shultz *et al.* (*4*), in which transitions were allowed from solitary to multimale and from multimale to pair living and to unimale and back. Transitions from solitary to social are not reversed, such that once a lineage becomes social, it remains so (Fig. 1C).

Last, we tested the model structure with the highest posterior support from the reversible-jump analysis described above. This model is similar to the IC model, but it also allows transitions from multimale to pair living and sets the transition from unimale to pair living equal to zero (Fig. 1D). A schematic representation of each model for the four-state scheme is reported in Fig. 1 (in fig. S1 for the three-state scheme). Each model was evaluated in five independent runs for 50 million iterations sampled every 1000 iterations, with the first 10 million iterations (20%) discarded as the burn-in period. Marginal likelihoods were calculated using stepping stone sampling with 100 samples and 10,000 iterations per sample. The stepping stone sampler estimates the marginal likelihood by placing a number of “stones,” which link the posterior with the prior; the stones are successively heated, forcing the chain from the posterior toward the prior. This procedure provides a more effective estimate of the marginal likelihood. Alternative models were then compared using log BFs

Log Bayes factors=2(log marginal likelihood[model 1] – log marginal likelihood [model 2])

The BF shows the weight of evidence to support one model over another (weak evidence, <2; positive evidence, >2; strong evidence, 5 to 10; very strong evidence, >10).

### Ancestral state reconstruction

Ancestral states were reconstructed using two approaches. First, we use the MRCA (most recent common ancestor) implemented in the MultiState package in the program BayesTraits 3.0 (*42*). We used BayesTraits to infer the posterior probability of alternative social systems at each ancestral node in the primate tree under the model with the highest posterior probability from the reversible-jump analysis (see above). The MCMC chain ran for 40 million iterations following a burn-in of 10 million iterations. The reversible-jump hyperprior values “exp (0 10)” were set to ensure adequate mixing and model acceptance rates.

Second, to estimate the marginal probabilities for all nodes based on joint sampling, stochastic mapping was implemented using the make.simmap function of the phytools package in R. Ancestral states were estimated using the ace command in the R package ape v3.4. We first performed stochastic character mapping with six different evolutionary models and compared these models using AIC scores. We used a continuous-time reversible Markov model fitted to our *Q* matrix (i.e., Q = “empirical”) and estimated the prior distribution on the root node of the tree based on tip character states (i.e., pi = “estimated”). We then calculated the corresponding AIC scores and AICw. After determining the AICw of each model, we ran 10,000 simulations of stochastic character mapping for the model that had the highest AICw. The average number of transitions between character states and the proportion of time spent in each state were summarized using phytools. Stochastic mapping using make.simmap does not allow polymorphic states. Therefore, to account for intraspecific variation, we coded traits in terms of probability (one state, *P* = 1.0; two states, *P*_1_ = *P*_2_ = 0.5; three states, *P*_1_ = *P*_2_ = *P*_3_ = 0.33). For instance, if a species was recorded as either solitary (S) or pair living (P), we coded the species as *P*_s_ = 0.5 and *P*_P_ = 0.5. This approach is not fully equivalent to account for the presence of intraspecific variability in social organization; however, it allowed us to incorporate a certain degree of uncertainty in tip states. Analyses were run using both known single states and a matrix of prior probabilities on tips.

### The influence of taxonomic sampling

To evaluate the impact of taxonomic sampling on our results, we randomly subsampled our original dataset (362 taxa) by creating 10 sampling schemes: 344 taxa (representing 95% of the taxa represented in the original dataset), 326 taxa (90%), 308 taxa (85%), 290 taxa (80%), 272 taxa (75%), 253 taxa (70%), 235 taxa (65%), 217 taxa (60%), 199 taxa (55%), and 181 taxa (50%). For each scheme, 10 independent subsamples were created and analyzed. For each subset, taxa were randomly selected from the original list of 362 species. Overall, a total of 100 datasets were run. For each of these 100 subsets, we explicitly tested the six models described above (ER, SYM, ARD, Shultz, RJ-MCMC, and IC) and identified the most likely model by comparing average AICw for each of the 10 independent subsamples.

## Supplementary Material

http://advances.sciencemag.org/cgi/content/full/5/12/eaay1276/DC1

Download PDF

Evolutionary transitions toward pair living in nonhuman primates as stepping stones toward more complex societies

## SUPPLEMENTARY MATERIALS

Supplementary material for this article is available at http://advances.sciencemag.org/cgi/content/full/5/12/eaay1276/DC1 Fig. S1. Alternative evolutionary models of social evolution for the three-state scheme. Fig. S2. Primate phylogeny showing ancestral state reconstructions for society under the IC model of evolution for the three-state scheme. Fig. S3. Results of the taxonomic sampling analyses. Table S1. Classification of social organization for the three-state scheme (S: solitary; P: pair living; G: group living) and the four-state scheme (S: solitary; P: pair living; UM: unimale groups; MM: multimale, multifemale groups) used in this study. Table S2. The *D* statistic for all binary traits. Table S3. Top 10 evolutionary models of primate social organization for the three-state scheme. Table S4. Top 10 evolutionary models of primate social organization for the four-state scheme. Table S5. Top 10 evolutionary models of primate social organization for the three-state scheme using 1000 different trees from the 10kTrees Project (version 3;59). Table S6. Top 10 evolutionary models of primate social organization for the four-state scheme using 1000 different trees from the 10kTrees project (version 3;59). Table S7. Average number of transitions inferred across 10,000 stochastic maps using SIMMAP function in R. Table S8. Proportion of pairs among primate social units with at least one pair.

## Appendix

View/request a protocol for this paper from *Bio-protocol*.
