# Supplementary material for: Evolutionary transitions toward pair living in nonhuman primates as stepping stones toward more complex societies
Source: Sci Adv. 2019 Dec 18;5(12):eaay1276. doi: 10.1126/sciadv.aay1276 (PMC6989303; doi:10.1126/sciadv.aay1276)
Supplement: http://advances.sciencemag.org/cgi/content/full/5/12/eaay1276/DC1 [file supp_5_12_eaay1276__index.html]

Science Advances | Science AdvancesAAASSearchScience AdvancesMenu

## Supplementary Materials

**This PDF file includes:**

- Fig. S1. Alternative evolutionary models of social evolution for the three-state scheme.
- Fig. S2. Primate phylogeny showing ancestral state reconstructions for society under the IC model of evolution for the three-state scheme.
- Fig. S3. Results of the taxonomic sampling analyses.
- Table S1. Classification of social organization for the three-state scheme (S: solitary; P: pair living; G: group living) and the four-state scheme (S: solitary; P: pair living; UM: unimale groups; MM: multimale, multifemale groups) used in this study.
- Table S2. The *D* statistic for all binary traits.
- Table S3. Top 10 evolutionary models of primate social organization for the three-state scheme.
- Table S4. Top 10 evolutionary models of primate social organization for the four-state scheme.
- Table S5. Top 10 evolutionary models of primate social organization for the three-state scheme using 1000 different trees from the 10kTrees Project (version 3;59).
- Table S6. Top 10 evolutionary models of primate social organization for the four-state scheme using 1000 different trees from the 10kTrees project (version 3;59).
- Table S7. Average number of transitions inferred across 10,000 stochastic maps using SIMMAP function in R.
- Table S8. Proportion of pairs among primate social units with at least one pair.

Download PDF

**Files in this Data Supplement:**

- Adobe PDF - aay1276\_SM.pdf
