# Supplementary material for: Evolutionary transitions toward pair living in nonhuman primates as stepping stones toward more complex societies
Source: Sci Adv. 2019 Dec 18;5(12):eaay1276. doi: 10.1126/sciadv.aay1276 (PMC6989303; doi:10.1126/sciadv.aay1276)
Supplement: Download PDF [file aay1276_SM.pdf]

Science Advances
AAAS

advances.sciencemag.org/cgi/content/full/5/12/eaay1276/DC1

Supplementary Materials for

Evolutionary transitions toward pair living in nonhuman primates as stepping stones toward more complex societies

Peter M. Kappeler* and Luca Pozzi

*Corresponding author. Email: pkappel@gwdg.de

Published 18 December 2019, *Sci. Adv.* **5**, eaay1276 (2019)
DOI: 10.1126/sciadv.aay1276

This PDF file includes:

Fig. S1. Alternative evolutionary models of social evolution for the three-state scheme.
Fig. S2. Primate phylogeny showing ancestral state reconstructions for society under the IC model of evolution for the three-state scheme.
Fig. S3. Results of the taxonomic sampling analyses.
Table S1. Classification of social organization for the three-state scheme (S: solitary; P: pair living; G: group living) and the four-state scheme (S: solitary; P: pair living; UM: unimale groups; MM: multimale, multifemale groups) used in this study.
Table S2. The *D* statistic for all binary traits.
Table S3. Top 10 evolutionary models of primate social organization for the three-state scheme.
Table S4. Top 10 evolutionary models of primate social organization for the four-state scheme.
Table S5. Top 10 evolutionary models of primate social organization for the three-state scheme using 1000 different trees from the 10kTrees Project (version 3;59).
Table S6. Top 10 evolutionary models of primate social organization for the four-state scheme using 1000 different trees from the 10kTrees project (version 3;59).
Table S7. Average number of transitions inferred across 10,000 stochastic maps using SIMMAP function in R.
Table S8. Proportion of pairs among primate social units with at least one pair.

SUPPLEMENTARY MATERIALS

Supplementary Figures

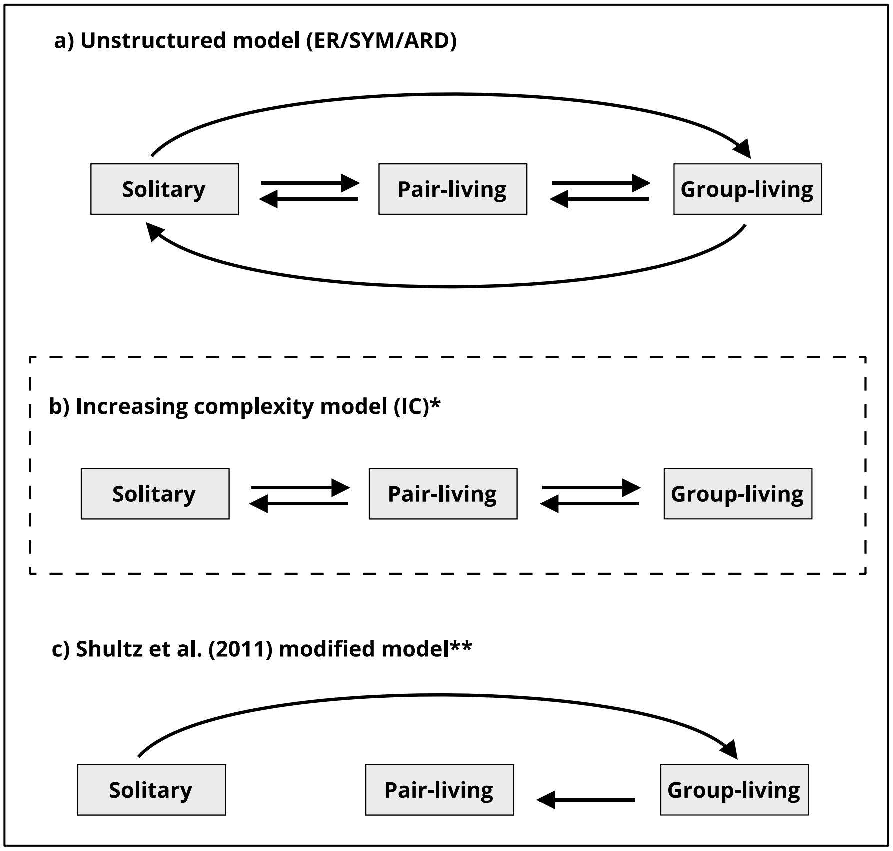

Fig. S1. Alternative evolutionary models of social evolution for the three-state scheme. Arrows represent permitted transitions between different social organizations for each model. (**a**) Unstructured models: under this model, all possible transitions are allowed. ER: all rates are fixed to a single optimized rate parameter; SYM: forward and reverse rates between two states are identical; ARD: rates are fully independent. (**b**) Increasing complexity model (IC): transitions are only allowed between solitary and pair living, pair living and uni-male groups, and uni-male groups and multi-male organization. This model is identical to the one selected by the Reversible-jump-derived model approach used in BayesTraits. (**c**) Model modified from Shultz et al. (*4*): transitions are allowed from solitary to group-living and then to pair living.
** The original model proposed by Shultz et al. (*4*) distinguished between uni-male and multi-male social structure (see Figure 1 in the manuscript).

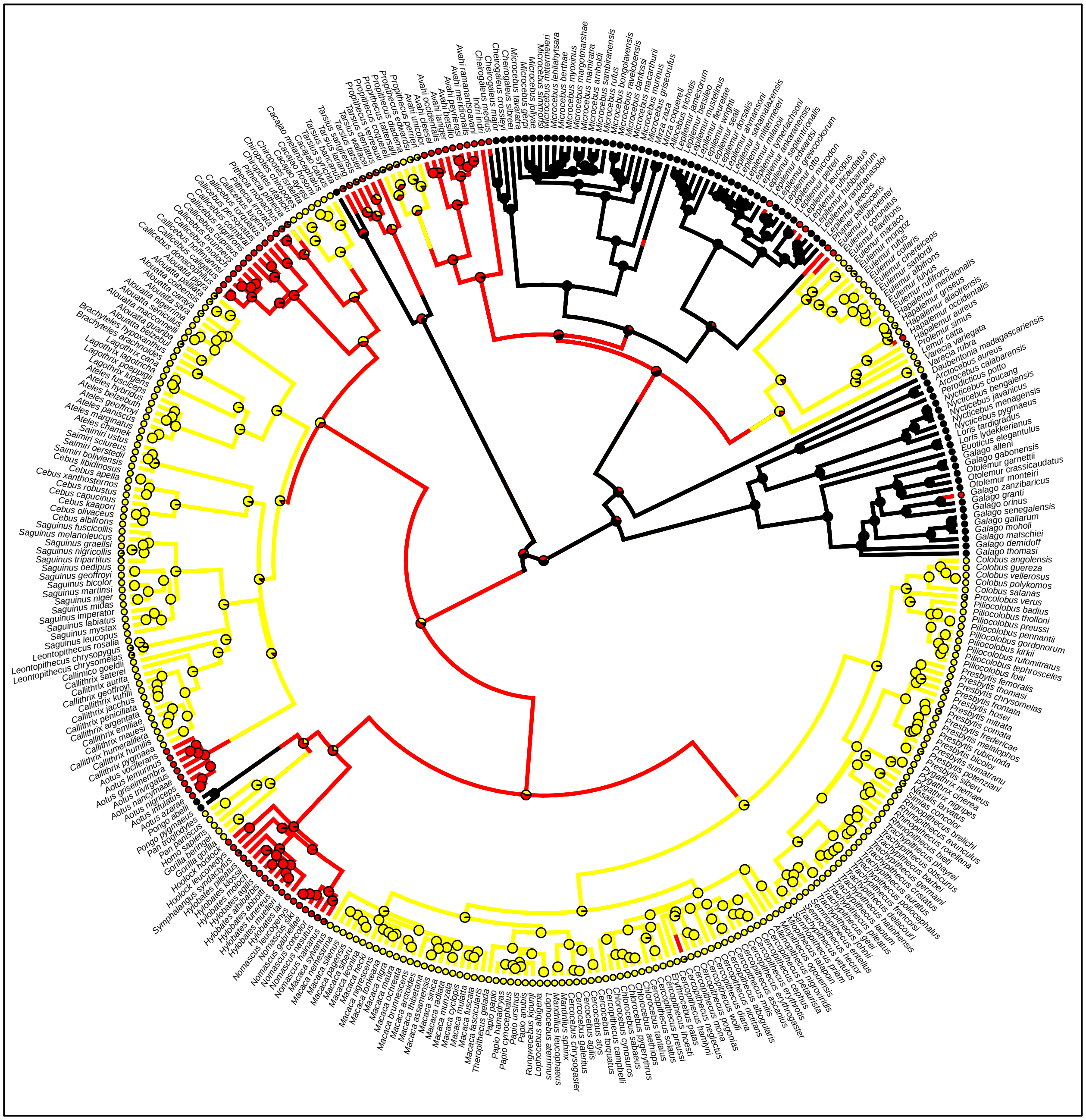

Fig. S2. Primate phylogeny showing ancestral state reconstructions for society under the IC model of evolution for the three-state scheme. Branches and tips are colored for solitary (black), pair living (red), group living (yellow) using one tree randomly selected from the 10,000 trees in the stochastic mapping process. Pies at each node are derived from ancestral state reconstruction using the function ace (R package 'ape').

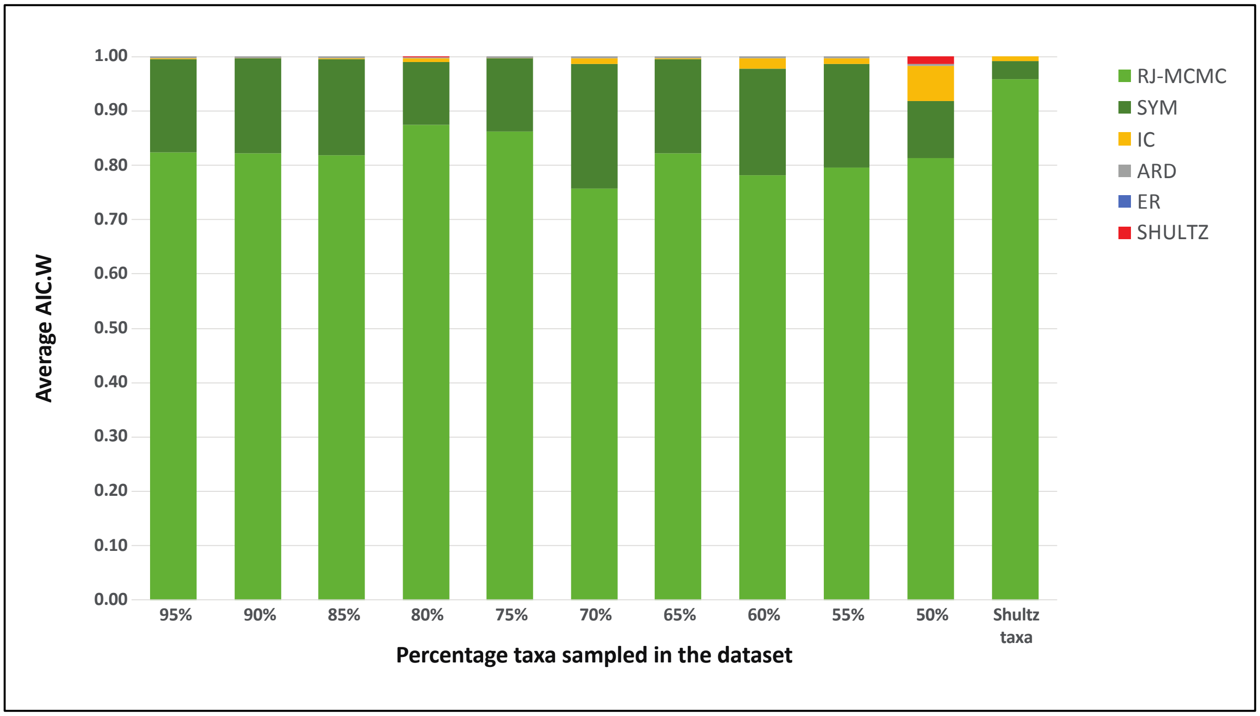

Fig. S3. Results of the taxonomic sampling analyses. The effect of taxonomic representation was tested across 11 different sampling schemes, all subsamples of the original dataset (362 taxa). The different sampling schemes were 344 taxa (representing 95% of the taxa represented in the original dataset), 326 taxa (90%), 308 taxa (85%), 290 taxa (80%), 272 taxa (75%), 253 taxa (70%), 235 taxa (65%), 217 taxa (60%), 199 taxa (55%) and 181 taxa (50%). Finally, we also tested the taxa used by Shultz et al. (*4*). Bar graphs represent average values for Akaike weights (AIC.w) for each tested across 10 independent runs.

Supplementary Tables

Table S1. Classification of social organization for the three-state scheme (S: solitary; P: pair living; G: group living) and the four-state scheme (S: solitary; P: pair living; UM: unimale groups; MM: multimale, multifemale groups) used in this study. We only include species that have actually been studied in the field (according to 38), ie we do not extrapolate from one or a few species to all members of a genus. We also present the coding used by Shultz et al. (4; "social polymorphism") and Lukas & Clutton-Brock (5) to illustrate the changes in sample size due to new field studies and taxonomy since their studies. Note that the latter studies include additional species that - according to (38) – have not yet been studied in the wild, however. Discrepancies in the coding of species for which two or three studies present data are mostly due to additional data available in the most recent source (38) and a different definition of pair living by (5), which determined the number of co-breeding females, whereas the other studies used the number of co-resident females.

Family	Species	3 states	4 states	(4)	(5)
Aotidae	Aotus azarae	P	P	P	P
Aotidae	Aotus griseimembra	P	P		
Aotidae	Aotus infulatus	P	P	P	
Aotidae	Aotus lemurinus	P	P	P	P
Aotidae	Aotus nancymaae	P	P	P	P
Aotidae	Aotus nigriceps	P	P		P
Aotidae	Aotus trivirgatus	P	P	P	P
Aotidae	Aotus vociferans	P	P		P
Atelidae	Alouatta belzebul	G	UM, MM		G
Atelidae	Alouatta caraya	P, G	P, UM, MM	UM, MM	G
Atelidae	Alouatta coibensis	G	UM, MM		G
Atelidae	Alouatta guariba	P, G	P, UM, MM		G
Atelidae	Alouatta macconnelli	G	UM, MM		G
Atelidae	Alouatta nigerrima	G	UM		G
Atelidae	Alouatta palliata	P, G	P, UM, MM	UM, MM	G
Atelidae	Alouatta pigra	P, G	P, UM, MM	UM, MM	G
Atelidae	Alouatta sara	G	UM	UM, MM	G
Atelidae	Alouatta seniculus	G	UM, MM	UM, MM	G
Atelidae	Ateles belzebuth	G	MM	MM	G
Atelidae	Ateles chamek	G	MM		G
Atelidae	Ateles fusciceps	G	MM		G
Atelidae	Ateles geoffroyi	G	MM	MM	G
Atelidae	Ateles hybridus	G	MM		G
Atelidae	Ateles marginatus	G	MM		G
Atelidae	Ateles paniscus	G	MM	MM	G
Atelidae	Brachyteles arachnoides	G	MM	MM	G
Atelidae	Brachyteles hypoxanthus	G	MM		G
Atelidae	Lagothrix cana	G	MM		G

Atelidae	Lagothrix lagotricha	G	MM	MM	G
Atelidae	Lagothrix lugens	G	MM		G
Atelidae	Lagothrix poeppigii	G	MM		G
Cebidae	Callimico goeldii	G	MM	P, UM, MM	G
Cebidae	Callithrix argentata	G	MM	P, UM, MM	P
Cebidae	Callithrix aurita	G	MM	P, UM, MM	P
Cebidae	Callithrix emiliae	G	MM	P, UM, MM	P
Cebidae	Callithrix geoffroyi	G	MM	P, UM, MM	P
Cebidae	Callithrix humeralifera	G	MM	P, UM, MM	P
Cebidae	Callithrix humilis	G	MM		
Cebidae	Callithrix jacchus	G	MM	P, UM, MM	P
Cebidae	Callithrix kuhlii	G	MM	P, UM, MM	P
Cebidae	Callithrix mauesi	G	MM		
Cebidae	Callithrix penicillata	G	MM	P, UM, MM	P
Cebidae	Callithrix pygmaea	G	UM	P, UM, MM	P
Cebidae	Callithrix saterei	G	MM		
Cebidae	Cebus albifrons	G	MM	MM	G
Cebidae	Cebus apella	G	MM	MM	G
Cebidae	Cebus capucinus	G	MM	MM	G
Cebidae	Cebus kaapori	G	MM		
Cebidae	Cebus libidinosus	G	MM		G
Cebidae	Cebus olivaceus	G	MM		G
Cebidae	Cebus robustus	G	MM		
Cebidae	Cebus xanthosternos	G	MM		G
Cebidae	Leontopithecus chrysomelas	P, G	P, UM, MM	P, UM, MM	P
Cebidae	Leontopithecus chrysopygus	P, G	P, MM	P, UM, MM	P
Cebidae	Leontopithecus rosalia	G	UM, MM	P, UM, MM	P
Cebidae	Saguinus bicolor	G	MM		P
Cebidae	Saguinus fuscicollis	G	UM, MM	P, UM, MM	P
Cebidae	Saguinus geoffroyi	G	MM	P, UM, MM	P
Cebidae	Saguinus graellsi	G	MM		P
Cebidae	Saguinus imperator	G	UM, MM	P, UM, MM	P
Cebidae	Saguinus labiatus	G	MM		P
Cebidae	Saguinus leucopus	G	MM		P
Cebidae	Saguinus martinsi	G	MM		P
Cebidae	Saguinus melanoleucus	G	MM		P
Cebidae	Saguinus midas	G	UM, MM	P, UM, MM	P
Cebidae	Saguinus mystax	G	UM, MM		P
Cebidae	Saguinus niger	G	MM		P
Cebidae	Saguinus nigricollis	P, G	P, MM		P

Cebidae	Saguinus oedipus	G	MM	P, UM, MM	P
Cebidae	Saguinus tripartitus	G	MM		P
Cebidae	Saimiri boliviensis	G	MM	MM	G
Cebidae	Saimiri oerstedii	G	MM	MM	G
Cebidae	Saimiri sciureus	G	MM	MM	G
Cebidae	Saimiri ustus	G	MM		G
Cercopithecidae	Allenopithecus nigroviridis	G	MM	MM	G
Cercopithecidae	Cercocebus agilis	G	UM, MM	MM	G
Cercopithecidae	Cercocebus atys	G	MM	MM	G
Cercopithecidae	Cercocebus chrysogaster	G	MM		G
Cercopithecidae	Cercocebus galeritus	G	UM, MM	MM	G
Cercopithecidae	Cercocebus torquatus	G	MM	MM	G
Cercopithecidae	Cercopithecus albogularis	G	UM		G
Cercopithecidae	Cercopithecus ascanius	G	UM	UM	G
Cercopithecidae	Cercopithecus campbelli	G	UM	UM	G
Cercopithecidae	Cercopithecus cephus	G	UM	UM	G
Cercopithecidae	Cercopithecus diana	G	UM	UM	G
Cercopithecidae	Cercopithecus erythrogaster	G	UM	UM	G
Cercopithecidae	Cercopithecus erythrotis	G	UM	UM	G
Cercopithecidae	Cercopithecus hamlyni	G	UM, MM	UM	G
Cercopithecidae	Cercopithecus lhoesti	G	UM	UM	G
Cercopithecidae	Cercopithecus mitis	G	UM	UM, MM	G
Cercopithecidae	Cercopithecus mona	G	UM, MM	UM	G
Cercopithecidae	Cercopithecus neglectus	P, G	P, UM	P, UM	G
Cercopithecidae	Cercopithecus nictitans	G	UM	UM	G
Cercopithecidae	Cercopithecus petaurista	G	UM	UM	G
Cercopithecidae	Cercopithecus pogonias	G	UM		G
Cercopithecidae	Cercopithecus preussi	G	UM, MM	UM	G
Cercopithecidae	Cercopithecus solatus	G	UM	UM	G
Cercopithecidae	Cercopithecus wolfi	G	UM, MM	UM	G
Cercopithecidae	Chlorocebus aethiops	G	MM	MM	G
Cercopithecidae	Chlorocebus cynosuros	G	MM		
Cercopithecidae	Chlorocebus pygerythrus	G	MM	MM	
Cercopithecidae	Chlorocebus sabaeus	G	MM	MM	G
Cercopithecidae	Chlorocebus tantalus	G	MM	MM	
Cercopithecidae	Colobus angolensis	G	MM	P, UM, MM	G
Cercopithecidae	Colobus guereza	G	MM	P, UM, MM	G
Cercopithecidae	Colobus polykomos	G	MM	UM, MM	G
Cercopithecidae	Colobus satanas	G	MM		G
Cercopithecidae	Colobus vellerosus	G	MM		G

Cercopithecidae	Erythrocebus patas	G	MM	UM	G
Cercopithecidae	Lophocebus albigena	G	MM	MM	G
Cercopithecidae	Lophocebus aterrimus	G	MM	MM	G
Cercopithecidae	Macaca arctoides	G	MM	MM	G
Cercopithecidae	Macaca assamensis	G	MM	MM	G
Cercopithecidae	Macaca brunnescens	G	MM	MM	
Cercopithecidae	Macaca cyclopis	G	MM	MM	G
Cercopithecidae	Macaca fascicularis	G	MM	MM	G
Cercopithecidae	Macaca fuscata	G	MM	MM	G
Cercopithecidae	Macaca hecki	G	MM	MM	G
Cercopithecidae	Macaca leonina	G	MM	MM	G
Cercopithecidae	Macaca maura	G	MM	MM	G
Cercopithecidae	Macaca mulatta	G	MM	MM	G
Cercopithecidae	Macaca munzala	G	MM		
Cercopithecidae	Macaca nemestrina	G	MM	MM	G
Cercopithecidae	Macaca nigra	G	MM	MM	G
Cercopithecidae	Macaca nigrescens	G	MM	MM	G
Cercopithecidae	Macaca ochreata	G	MM	MM	G
Cercopithecidae	Macaca pagensis	G	MM	UM	G
Cercopithecidae	Macaca radiata	G	MM	MM	G
Cercopithecidae	Macaca siberu	G	MM	MM	
Cercopithecidae	Macaca silenus	G	UM	MM	G
Cercopithecidae	Macaca sinica	G	MM	MM	G
Cercopithecidae	Macaca sylvanus	G	MM	MM	G
Cercopithecidae	Macaca thibetana	G	MM	MM	G
Cercopithecidae	Macaca tonkeana	G	MM	MM	G
Cercopithecidae	Mandrillus leucophaeus	G	UM, MM	MM	G
Cercopithecidae	Mandrillus sphinx	G	UM, MM	MM	G
Cercopithecidae	Miopithecus ogouensis	G	MM		
Cercopithecidae	Miopithecus talapoin	G	MM	MM	G
Cercopithecidae	Nasalis larvatus	P, G	P, UM	UM	G
Cercopithecidae	Papio anubis	G	MM	MM	G
Cercopithecidae	Papio cynocephalus	G	MM	MM	G
Cercopithecidae	Papio hamadryas	G	UM	UM, MM	G
Cercopithecidae	Papio papio	G	MM	MM	G
Cercopithecidae	Papio ursinus	G	MM	MM	G
Cercopithecidae	Piliocolobus badius	G	MM	MM	
Cercopithecidae	Piliocolobus foai	G	MM		
Cercopithecidae	Piliocolobus gordonorum	G	MM		G
Cercopithecidae	Piliocolobus kirkii	G	MM		G

Cercopithecidae	Piliocolobus pennantii	G	UM, MM		G
Cercopithecidae	Piliocolobus preussi	G	MM		G
Cercopithecidae	Piliocolobus rufomitratus	G	MM		G
Cercopithecidae	Piliocolobus tephrosceles	G	MM		G
Cercopithecidae	Piliocolobus tholloni	G	MM		G
Cercopithecidae	Presbytis bicolor	G	UM		
Cercopithecidae	Presbytis chrysomelas	G	UM		G
Cercopithecidae	Presbytis comata	P, G	P, UMMM		G
Cercopithecidae	Presbytis femoralis	G	UM		G
Cercopithecidae	Presbytis fredericae	G	UM		
Cercopithecidae	Presbytis frontata	P, G	P, UM		G
Cercopithecidae	Presbytis hosei	G	UM		G
Cercopithecidae	Presbytis melalophos	G	UM, MM	UM, MM	G
Cercopithecidae	Presbytis mitrata	G	UM		
Cercopithecidae	Presbytis potenziani	P, G	P, UM, MM		G
Cercopithecidae	Presbytis rubicunda	G	UM		G
Cercopithecidae	Presbytis siberu	G	UM		
Cercopithecidae	Presbytis sumatranu	G	UM		
Cercopithecidae	Presbytis thomasi	G	UM, MM		G
Cercopithecidae	Procolobus verus	P, G	P, UM, MM		G
Cercopithecidae	Pygathrix cinerea	G	UM, MM		
Cercopithecidae	Pygathrix nemaeus	G	UM, MM	UM, MM	G
Cercopithecidae	Pygathrix nigripes	G	UM		
Cercopithecidae	Rhinopithecus avunculus	G	UM, MM	UM	
Cercopithecidae	Rhinopithecus bieti	G	UM	UM	
Cercopithecidae	Rhinopithecus brelichi	G	UM, MM	UM	
Cercopithecidae	Rhinopithecus roxellana	G	UM	UM	
Cercopithecidae	Rungwecebus kipunji	G	MM	MM	
Cercopithecidae	Semnopithecus entellus	G	UM, MM	UM, MM	G
Cercopithecidae	Semnopithecus hector	G	UM, MM		G
Cercopithecidae	Semnopithecus priam	G	UM, MM		G
Cercopithecidae	Simias concolor	P, G	P, UM		G
Cercopithecidae	Theropithecus gelada	G	UM, MM	UM	
Cercopithecidae	Trachypithecus auratus	G	UM	UM	G
Cercopithecidae	Trachypithecus barbei	G	UM		G
Cercopithecidae	Trachypithecus cristatus	G	UM, MM	MM	G
Cercopithecidae	Trachypithecus delacouri	G	UM		G
Cercopithecidae	Trachypithecus francoisi	G	UM	UM	G
Cercopithecidae	Trachypithecus geei	G	UM, MM		G
Cercopithecidae	Trachypithecus germaini	G	MM		G

Cercopithecidae	Trachypithecus hatinhensis	G	UM		G
Cercopithecidae	Trachypithecus johnii	G	MM	UM, MM	G
Cercopithecidae	Trachypithecus laotum	G	UM		G
Cercopithecidae	Trachypithecus obscurus	G	UM, MM	MM	G
Cercopithecidae	Trachypithecus phayrei	G	UM, MM	UM	G
Cercopithecidae	Trachypithecus pileatus	G	UM, MM	UM	G
Cercopithecidae	Trachypithecus poliocephalus	G	UM	UM	G
Cercopithecidae	Trachypithecus vetulus	G	UM		G
Cheirogaleidae	Allocebus trichotis	S	S	S, P	S
Cheirogaleidae	Cheirogaleus crossleyi	S	S		P
Cheirogaleidae	Cheirogaleus major	S	S	S	P
Cheirogaleidae	Cheirogaleus medius	P	P	S, P	P
Cheirogaleidae	Cheirogaleus sibreei	S	S		P
Cheirogaleidae	Microcebus arnholdi	S	S		
Cheirogaleidae	Microcebus berthae	S	S		
Cheirogaleidae	Microcebus bongolavensis	S	S		
Cheirogaleidae	Microcebus danfossi	S	S		
Cheirogaleidae	Microcebus gerpi	S	S		
Cheirogaleidae	Microcebus griseorufus	S	S		S
Cheirogaleidae	Microcebus jollyae	S	S		
Cheirogaleidae	Microcebus lehilahytsara	S	S		
Cheirogaleidae	Microcebus macarthurii	S	S		
Cheirogaleidae	Microcebus mamiratra	S	S		
Cheirogaleidae	Microcebus margotmarshae	S	S		
Cheirogaleidae	Microcebus mittermeieri	S	S		
Cheirogaleidae	Microcebus murinus	S	S	S, MM	G
Cheirogaleidae	Microcebus myoxinus	S	S	S	S
Cheirogaleidae	Microcebus ravelobensis	S	S	S, MM	
Cheirogaleidae	Microcebus rufus	S	S	S	S
Cheirogaleidae	Microcebus sambiranensis	S	S		
Cheirogaleidae	Microcebus simmonsi	S	S		
Cheirogaleidae	Microcebus tavaratra	S	S		
Cheirogaleidae	Mirza coquereli	S	S	S	S
Cheirogaleidae	Mirza zaza	S	S		
Cheirogaleidae	Phaner pallescens	P	P		P
Daubentoniidae	Daubentonia madagascariensis	S	S	S	S
Galagidae	Euoticus elegantulus	S	S	S, UM	S
Galagidae	Galago alleni	S	S	S, UM	S
Galagidae	Galago demidoff	S	S	S, P, UM	S
Galagidae	Galago gabonensis	S	S		S

Galagidae	Galago gallarum	S	S	S	S
Galagidae	Galago granti	S	S		S
Galagidae	Galago matschiei	S	S		S
Galagidae	Galago moholi	S	S	S, UM	S
Galagidae	Galago orinus	S	S		S
Galagidae	Galago senegalensis	S	S	S, UM	S
Galagidae	Galago thomasi	S	S		S
Galagidae	Galago zanzibaricus	P	P	S, P, UM	S
Galagidae	Otolemur crassicaudatus	S	S	S, P, UM	S
Galagidae	Otolemur garnettii	S	S	S	S
Galagidae	Otolemur monteiri	S	S		S
Hominidae	Gorilla beringei	G	UM, MM		G
Hominidae	Gorilla gorilla	G	UM	UM, MM	G
Hominidae	Homo sapiens	G	MM	MM	
Hominidae	Pan paniscus	G	MM	MM	G
Hominidae	Pan troglodytes	G	MM	MM	G
Hominidae	Pongo abelii	S	S	S, MM	S
Hominidae	Pongo pygmaeus	S	S	S, MM	S
Hylobatidae	Hoolock hoolock	P	P	P	P
Hylobatidae	Hoolock leuconedys	P	P		
Hylobatidae	Hylobates abbotti	P	P		
Hylobatidae	Hylobates agilis	P	P	P	P
Hylobatidae	Hylobates albibarbis	P	P		
Hylobatidae	Hylobates funereus	P	P		
Hylobatidae	Hylobates klossii	P, G	P, MM	P	
Hylobatidae	Hylobates lar	P	P	P	P
Hylobatidae	Hylobates moloch	P	P	P	P
Hylobatidae	Hylobates muelleri	P	P	P	P
Hylobatidae	Hylobates pileatus	P	P	P	P
Hylobatidae	Nomascus concolor	P, G	P, UM	P	
Hylobatidae	Nomascus gabriellae	P	P	P	
Hylobatidae	Nomascus hainanus	P, G	P, UM		
Hylobatidae	Nomascus leucogenys	P	P	P	
Hylobatidae	Nomascus nasutus	P, G	P, UM		
Hylobatidae	Nomascus siki	P	P		
Hylobatidae	Symphalangus syndactylus	P, G	P, MM	P	P
Indriidae	Avahi betsilio	P	P		
Indriidae	Avahi cleesei	P	P		
Indriidae	Avahi laniger	P	P	P	P
Indriidae	Avahi meridionalis	P	P		

Indriidae	Avahi occidentalis	P	P	P	P
Indriidae	Avahi peyrierasi	P	P		
Indriidae	Avahi ramanantsoavani	P	P		
Indriidae	Avahi unicolor	P	P		
Indriidae	Indri indri	P	P	P	P
Indriidae	Propithecus coquereli	P, G	P, UM, MM	P, UM, MM	G
Indriidae	Propithecus diadema	G	UM, MM	P, UM, MM	G
Indriidae	Propithecus edwardsi	P, G	P, UM, MM	P, UM, MM	G
Indriidae	Propithecus perrieri	P, G	P, MM		G
Indriidae	Propithecus tattersalli	P, G	P, UM, MM	MM	G
Indriidae	Propithecus verreauxi	P, G	P, UM, MM	P, UM, MM	G
Lemuridae	Eulemur albifrons	G	MM	MM	
Lemuridae	Eulemur cinereiceps	G	MM		
Lemuridae	Eulemur collaris	G	MM	MM	
Lemuridae	Eulemur coronatus	G	MM	MM	G
Lemuridae	Eulemur flavifrons	G	MM	MM	
Lemuridae	Eulemur fulvus	G	MM	MM	G
Lemuridae	Eulemur macaco	P, G	P, UM, MM	MM	G
Lemuridae	Eulemur mongoz	P, G	P, MM	P, MM	P
Lemuridae	Eulemur rubriventer	P	P	P	P
Lemuridae	Eulemur rufifrons	G	MM	MM	
Lemuridae	Eulemur rufus	G	MM		G
Lemuridae	Eulemur sanfordi	G	MM	MM	
Lemuridae	Hapalemur alaotrensis	G	MM	P	G
Lemuridae	Hapalemur aureus	P, G	P, UM	P, MM	G
Lemuridae	Hapalemur griseus	P, G	P, UM	P	G
Lemuridae	Hapalemur meridionalis	G	MM	P	
Lemuridae	Hapalemur occidentalis	P	P	P	G
Lemuridae	Lemur catta	G	MM	MM	G
Lemuridae	Prolemur simus	G	UM, MM	MM	G
Lemuridae	Varecia rubra	G	MM	MM	G
Lemuridae	Varecia variegata	P, G	P, MM	MM	G
Lepilemuridae	Lepilemur aeeclis	S	S	S	
Lepilemuridae	Lepilemur ahmansoni	S	S		
Lepilemuridae	Lepilemur ankaranensis	S	S		
Lepilemuridae	Lepilemur betsileo	S	S		
Lepilemuridae	Lepilemur dorsalis	S	S	S	S
Lepilemuridae	Lepilemur edwardsi	P	P	S	S
Lepilemuridae	Lepilemur fleuretae	S	S		
Lepilemuridae	Lepilemur grewcockorum	S	S		

Lepilemuridae	Lepilemur hubbardorum	S	S		
Lepilemuridae	Lepilemur jamesorum	S	S		
Lepilemuridae	Lepilemur leucopus	P	P	S	S
Lepilemuridae	Lepilemur microdon	S	S	S	S
Lepilemuridae	Lepilemur milanoii	S	S		
Lepilemuridae	Lepilemur mittermeieri	S	S		
Lepilemuridae	Lepilemur mustelinus	S	S	S	S
Lepilemuridae	Lepilemur otto	S	S		
Lepilemuridae	Lepilemur petteri	S	S		
Lepilemuridae	Lepilemur randrianasoloi	S	S	S	
Lepilemuridae	Lepilemur ruficaudatus	P	P	S	S
Lepilemuridae	Lepilemur sahamalazensis	S	S	S	
Lepilemuridae	Lepilemur seali	S	S	S	
Lepilemuridae	Lepilemur septentrionalis	S	S	S	S
Lepilemuridae	Lepilemur tymerlachsoni	S	S		
Lepilemuridae	Lepilemur wrighti	S	S		
Lorisidae	Arctocebus aureus	S	S		
Lorisidae	Arctocebus calabarensis	S	S	S	S
Lorisidae	Loris lydekkerianus	S	S	S, P	S
Lorisidae	Loris tardigradus	S	S	S, P	S
Lorisidae	Nycticebus bengalensis	S	S		S
Lorisidae	Nycticebus coucang	S	S	S	S
Lorisidae	Nycticebus javanicus	S	S		
Lorisidae	Nycticebus menagensis	S	S		
Lorisidae	Nycticebus pygmaeus	S	S	S	S
Lorisidae	Perodicticus potto	S	S	S	S
Pitheciidae	Cacajao ayresi	G	MM		
Pitheciidae	Cacajao calvus	G	MM		G
Pitheciidae	Cacajao hosomi	G	MM		
Pitheciidae	Cacajao melanocephalus	G	MM		G
Pitheciidae	Callicebus brunneus	P	P		P
Pitheciidae	Callicebus caligatus	P	P		
Pitheciidae	Callicebus coimbrai	P	P		
Pitheciidae	Callicebus cupreus	P	P		P
Pitheciidae	Callicebus donacophilus	P	P	P	P
Pitheciidae	Callicebus hoffmannsi	P	P		P
Pitheciidae	Callicebus lugens	P	P		P
Pitheciidae	Callicebus moloch	P	P	P	P
Pitheciidae	Callicebus nigrifrons	P	P		P
Pitheciidae	Callicebus personatus	P	P		P

Pitheciidae	Callicebus torquatus	P	P		P
Pitheciidae	Chiropotes chiropotes	G	MM		G
Pitheciidae	Chiropotes israelita	G	MM		G
Pitheciidae	Chiropotes utahicki	G	MM		G
Pitheciidae	Pithecia irrorata	P	P		P
Pitheciidae	Pithecia monachus	P, G	P, MM		P
Pitheciidae	Pithecia pithecia	P, G	P, MM		P
Tarsiidae	Tarsius bancanus	P, G	P, UM	S	S
Tarsiidae	Tarsius dentatus	P, G	P, UM		S
Tarsiidae	Tarsius lariang	P, G	P, UM		
Tarsiidae	Tarsius sangirensis	P, G	P, MM		S
Tarsiidae	Tarsius syrichta	S	S	S	S
Tarsiidae	Tarsius tarsier	P, G	P, UM		S
Tarsiidae	Tarsius wallacei	P, G	P, UM		

Table S2. The *D* statistic for all binary traits. The non-significant p-values are in bold, which means the traits are under Brownian evolution

3-state scheme (S-P-G)			
	Social structure		
	Solitary (S)	Pair living (P)	Group living (G)
Estimated *D*	-0.261	-0.094	-0.270
p random model	<0.01	<0.01	<0.01
p Brownian model	**0.961**	**0.738**	**0.981**

4-state scheme (S-P-UM-MM)				
	Social structure			
	Solitary (S)	Pair living (P)	Uni-male units (UM)	Multi-male multi-female units (MM)
Estimated *D*	-0.261	-0.094	0.139	0.004
p random model	<0.01	<0.01	<0.01	<0.01
p Brownian model	**0.961**	**0.738**	**0.204**	**0.490**

Table S3. Top 10 evolutionary models of primate social organization for the three-state scheme. Independently from the hyperpriors used in model selection analyses, the top ten models account for >90% of the posterior sample. The most frequent model is the same across all the different hyperpriors employed. Z = zero. 0s and 1s indicate distinct non-zero transition rates. Subscripts on the rate coefficients correspond to the transitions between the four states of social organization (0: solitary; 1: pair living; 2: group living). Frequency = visits to the model in the posterior distribution of 75,000.

**Priors reverse jump for model selection: "rjhp gamma 0 10 0 10"**								
**Model**	**Transition rates**						**Frequency**	**Cumulative frequency**
	q_{01}	q_{02}	q_{10}	q_{12}	q_{20}	q_{21}		
1	0	Z	0	0	Z	0	0.78	0.78
2	0	0	0	0	Z	0	0.06	0.85
3	0	0	0	Z	Z	0	0.02	0.87
4	0	Z	0	0	Z	Z	0.02	0.89
5	0	Z	Z	0	0	0	0.02	0.90
6	0	0	Z	0	Z	0	0.02	0.92
7	0	Z	0	0	0	0	0.01	0.93
8	0	Z	0	Z	Z	0	0.01	0.94
9	0	Z	Z	Z	0	0	0.01	0.96
10	0	Z	0	Z	0	0	0.01	0.96

**Priors reverse jump for model selection: "rj uniform -100 100"**								
**Model**	**Transition rates**						**Frequency**	**Cumulative frequency**
	q_{01}	q_{02}	q_{10}	q_{12}	q_{20}	q_{21}		
1	0	Z	0	0	Z	0	0.77	0.77
2	0	0	0	0	Z	0	0.06	0.83
3	0	Z	0	0	Z	Z	0.02	0.86
4	0	0	0	Z	Z	0	0.02	0.87
5	0	Z	Z	0	0	0	0.01	0.89
6	0	0	Z	0	Z	0	0.01	0.90
7	0	Z	0	0	0	0	0.01	0.91
8	0	Z	0	Z	Z	0	0.01	0.92
9	0	Z	Z	Z	0	0	0.01	0.93
10	0	Z	0	Z	0	0	0.01	0.94

**Priors reverse jump for model selection: "rjhp = exp 0 100"**								
**Model**	**Transition rates**						**Frequency**	**Cumulative frequency**
	q_{01}	q_{02}	q_{10}	q_{12}	q_{20}	q_{21}		
1	0	Z	0	0	Z	0	0.79	0.79
2	0	0	0	0	Z	0	0.06	0.86
3	0	0	0	Z	Z	0	0.02	0.88
4	0	Z	0	0	Z	Z	0.02	0.90
5	0	Z	Z	0	0	0	0.02	0.91
6	0	0	Z	0	Z	0	0.02	0.93
7	0	Z	0	0	0	0	0.01	0.94
8	0	Z	Z	Z	0	0	0.01	0.95
9	0	Z	0	Z	Z	0	0.01	0.97
10	0	Z	0	Z	0	0	0.01	0.97

Priors reverse jump for model selection: “rjhp exp 10”								
**Model**	**Transition rates**						**Frequency**	**Cumulative frequency**
	q_{01}	q_{02}	q_{10}	q_{12}	q_{20}	q_{21}		
1	0	Z	0	0	Z	0	0.78	0.78
2	0	0	0	0	Z	0	0.07	0.85
3	0	0	0	Z	Z	0	0.02	0.87
4	0	Z	0	0	Z	Z	0.02	0.88
5	0	Z	Z	0	0	0	0.02	0.90
6	0	0	Z	0	Z	0	0.02	0.92
7	0	Z	0	0	0	0	0.01	0.93
8	0	Z	Z	Z	0	0	0.01	0.95
9	0	Z	0	Z	Z	0	0.01	0.96
10	0	Z	0	Z	0	0	0.01	0.96

Priors reverse jump for model selection: “rjhp = exp 0 1"								
**Model**	**Transition rates**						**Frequency**	**Cumulative frequency**
	q_{01}	q_{02}	q_{10}	q_{12}	q_{20}	q_{21}		
1	0	Z	0	0	Z	0	0.71	0.71
2	0	0	0	0	Z	0	0.09	0.80
3	0	Z	Z	0	0	0	0.03	0.83
4	0	0	0	Z	Z	0	0.02	0.85
5	0	Z	0	0	0	0	0.02	0.88
6	0	0	Z	0	Z	0	0.02	0.90
7	0	Z	Z	Z	0	0	0.02	0.92
8	0	Z	0	Z	0	0	0.01	0.93
9	0	0	Z	0	0	0	0.01	0.94
10	0	0	Z	Z	Z	0	0.01	0.95

Table S4. Top 10 evolutionary models of primate social organization for the four-state scheme. Independently from the hyperpriors used in model selection analyses, the top ten models account for >90% of the posterior sample. The most frequent model is the same across all the different hyperpriors employed. Z = zero. 0s and 1s indicate distinct non-zero transition rates. Subscripts on the rate coefficients correspond to the transitions between the four states of social organization (0: solitary; 1: pair living; 2: uni-male, 3: multi-male). Frequency = visits to the model in the posterior distribution of 75,000.

**Priors reverse jump for model selection: "rjhp gamma 0 10 0 10"**														
**Model**	**Transition rates**												**Frequency**	**Cumulative frequency**
	q_{01}	q_{02}	q_{03}	q_{10}	q_{12}	q_{13}	q_{20}	q_{21}	q_{23}	q_{30}	q_{31}	q_{32}		
1	0	Z	Z	0	Z	0	Z	Z	0	Z	0	0	0.79	0.79
2	0	Z	Z	0	Z	0	0	Z	0	Z	0	0	0.10	0.89
3	0	Z	Z	0	Z	0	0	0	0	Z	0	0	0.02	0.91
4	0	Z	Z	0	0	0	Z	Z	0	Z	0	0	0.02	0.93
5	0	Z	Z	0	Z	0	0	Z	0	Z	Z	0	0.01	0.94
6	0	Z	Z	Z	Z	0	0	0	0	Z	0	0	0.01	0.94
7	0	Z	Z	0	0	0	Z	0	0	Z	0	0	0.01	0.95
8	0	Z	Z	Z	0	0	0	Z	0	Z	0	0	0.00	0.95
9	0	Z	0	0	Z	0	Z	Z	0	Z	0	0	0.00	0.96
10	0	Z	Z	0	0	0	0	Z	0	Z	0	0	0.00	0.96

**Priors reverse jump for model selection: "rj uniform -100 100"**														
**Model**	**Transition rates**												**Frequency**	**Cumulative frequency**
	q_{01}	q_{02}	q_{03}	q_{10}	q_{12}	q_{13}	q_{20}	q_{21}	q_{23}	q_{30}	q_{31}	q_{32}		
1	0	Z	Z	0	Z	0	Z	Z	0	Z	0	0	0.77	0.77
2	0	Z	Z	0	Z	0	0	Z	0	Z	0	0	0.09	0.87
3	0	Z	Z	0	Z	0	0	0	0	Z	0	0	0.02	0.89
4	0	Z	Z	0	0	0	Z	Z	0	Z	0	0	0.02	0.91
5	0	Z	Z	Z	Z	0	0	0	0	Z	0	0	0.01	0.91
6	0	Z	Z	0	Z	0	0	Z	0	Z	Z	0	0.01	0.92
7	0	Z	Z	0	0	0	Z	0	0	Z	0	0	0.01	0.92
8	0	Z	Z	Z	0	0	0	Z	0	Z	0	0	0.00	0.93
9	0	Z	Z	0	0	0	0	Z	0	Z	0	0	0.00	0.93
10	0	Z	0	0	Z	0	Z	Z	0	Z	0	0	0.00	0.93

**Priors reverse jump for model selection: "rjhp = exp 0 100"**														
**Model**	**Transition rates**												**Frequency**	**Cumulative frequency**
	q_{01}	q_{02}	q_{03}	q_{10}	q_{12}	q_{13}	q_{20}	q_{21}	q_{23}	q_{30}	q_{31}	q_{32}		
1	0	Z	Z	0	Z	0	Z	Z	0	Z	0	0	0.80	0.80
2	0	Z	Z	0	Z	0	0	Z	0	Z	0	0	0.10	0.90
3	0	Z	Z	0	Z	0	0	0	0	Z	0	0	0.02	0.92
4	0	Z	Z	0	0	0	Z	Z	0	Z	0	0	0.02	0.94
5	0	Z	Z	Z	Z	0	0	0	0	Z	0	0	0.01	0.95
6	0	Z	Z	0	Z	0	0	Z	0	Z	Z	0	0.01	0.95
7	0	Z	Z	0	0	0	Z	0	0	Z	0	0	0.01	0.96
8	0	Z	Z	0	0	0	0	Z	0	Z	0	0	0.00	0.96
9	0	Z	0	0	Z	0	Z	Z	0	Z	0	0	0.00	0.97
10	0	Z	Z	Z	0	0	0	Z	0	Z	0	0	0.00	0.97

Priors reverse jump for model selection: “rjhp exp 10”														
**Model**	**Transition rates**												**Frequency**	**Cumulative frequency**
	q_{01}	q_{02}	q_{03}	q_{10}	q_{12}	q_{13}	q_{20}	q_{21}	q_{23}	q_{30}	q_{31}	q_{32}		
1	0	Z	Z	0	Z	0	Z	Z	0	Z	0	0	0.79	0.79
2	0	Z	Z	0	Z	0	0	Z	0	Z	0	0	0.10	0.89
3	0	Z	Z	0	Z	0	0	0	0	Z	0	0	0.02	0.91
4	0	Z	Z	0	0	0	Z	Z	0	Z	0	0	0.02	0.93
5	0	Z	Z	Z	Z	0	0	0	0	Z	0	0	0.01	0.94
6	0	Z	Z	0	0	0	Z	0	0	Z	0	0	0.01	0.94
7	0	Z	Z	0	Z	0	0	Z	0	Z	Z	0	0.01	0.95
8	0	Z	Z	0	0	0	0	Z	0	Z	0	0	0.00	0.95
9	0	Z	Z	Z	0	0	0	Z	0	Z	0	0	0.00	0.95
10	0	Z	0	0	Z	0	Z	Z	0	Z	0	0	0.00	0.96

Priors reverse jump for mode lselection: “rjhp = exp 0 1"														
**Model**	**Transition rates**												**Frequency**	**Cumulative frequency**
	q_{01}	q_{02}	q_{03}	q_{10}	q_{12}	q_{13}	q_{20}	q_{21}	q_{23}	q_{30}	q_{31}	q_{32}		
1	0	Z	Z	0	Z	0	Z	Z	0	Z	0	0	0.76	0.76
2	0	Z	Z	0	Z	0	0	Z	0	Z	0	0	0.11	0.87
3	0	Z	Z	0	Z	0	0	0	0	Z	0	0	0.03	0.90
4	0	Z	Z	0	0	0	Z	Z	0	Z	0	0	0.02	0.92
5	0	Z	Z	0	0	0	Z	0	0	Z	0	0	0.01	0.92
6	0	Z	Z	Z	Z	0	0	0	0	Z	0	0	0.01	0.93
7	0	Z	Z	0	0	0	0	Z	0	Z	0	0	0.01	0.94
8	0	Z	0	0	Z	0	Z	Z	0	Z	0	0	0.01	0.94
9	0	Z	Z	Z	0	0	0	Z	0	Z	0	0	0.00	0.95
10	0	Z	Z	0	Z	0	0	Z	0	Z	Z	0	0.00	0.95

Table S5. Top 10 evolutionary models of primate social organization for the three-state scheme using 1000 different trees from the 10kTrees Project (version 3;59). Independently from the hyperpriors used in model selection analyses, the top ten models account for >90% of the posterior sample. The most frequent model is the same across all the different hyperpriors employed. Z = zero. 0s and 1s indicate distinct non-zero transition rates. Subscripts on the rate coefficients correspond to the transitions between the four states of social organization (0: solitary; 1: pair living; 2: group living). Frequency = visits to the model in the posterior distribution of 75,000.

Priors reverse jump for model selection: "rjhp gamma 0 10 0 10"								
**Model**	**Transition rates**						**Frequency**	**Cumulative frequency**
	q_{01}	q_{02}	q_{10}	q_{12}	q_{20}	q_{21}		
1	0	Z	0	0	Z	0	0.81	0.81
2	0	0	0	0	Z	0	0.10	0.91
3	0	0	0	Z	Z	0	0.02	0.93
4	0	0	Z	0	Z	0	0.02	0.95
5	0	Z	0	0	0	0	0.01	0.96
6	0	Z	Z	0	0	0	0.01	0.97
7	0	0	Z	Z	Z	0	0.01	0.97
8	0	Z	0	Z	Z	0	0.01	0.98
9	0	0	Z	0	0	0	0.00	0.98
10	0	Z	Z	Z	0	0	0.00	0.99

Priors reverse jump for model selection: "rj uniform -100 100"								
**Model**	**Transition rates**						**Frequency**	**Cumulative frequency**
	q_{01}	q_{02}	q_{10}	q_{12}	q_{20}	q_{21}		
1	0	Z	0	0	Z	0	0.83	0.83
2	0	0	0	0	Z	0	0.09	0.93
3	0	0	0	Z	Z	0	0.02	0.95
4	0	0	Z	0	Z	0	0.02	0.96
5	0	Z	0	0	0	0	0.01	0.97
6	0	Z	0	Z	Z	0	0.01	0.98
7	0	0	Z	Z	Z	0	0.01	0.98
8	0	Z	Z	0	0	0	0.01	0.99
9	0	0	Z	0	0	0	0.00	0.99
10	0	Z	Z	Z	0	0	0.00	0.99

Priors reverse jump for model selection: "rjhp = exp 0 100"								
**Model**	**Transition rates**						**Frequency**	**Cumulative frequency**
	q_{01}	q_{02}	q_{10}	q_{12}	q_{20}	q_{21}		
1	0	Z	0	0	Z	0	0.83	0.83
2	0	0	0	0	Z	0	0.10	0.92
3	0	0	0	Z	Z	0	0.02	0.94
4	0	0	Z	0	Z	0	0.02	0.96
5	0	Z	0	0	0	0	0.01	0.97
6	0	Z	0	Z	Z	0	0.01	0.97
7	0	Z	Z	0	0	0	0.01	0.98
8	0	0	Z	Z	Z	0	0.00	0.98
9	0	0	Z	0	0	0	0.00	0.99
10	0	Z	Z	Z	0	0	0.00	0.99

Priors reverse jump for model selection: “rjhp exp 10”								
Model	Transition rates						Frequency	Cumulative frequency
	q_{01}	q_{02}	q_{10}	q_{12}	q_{20}	q_{21}		
1	0	Z	0	0	Z	0	0.83	0.83
2	0	0	0	0	Z	0	0.09	0.92
3	0	0	Z	0	Z	0	0.02	0.94
4	0	0	0	Z	Z	0	0.02	0.96
5	0	Z	0	0	0	0	0.01	0.97
6	0	Z	0	Z	Z	0	0.01	0.97
7	0	0	Z	Z	Z	0	0.01	0.98
8	0	Z	Z	0	0	0	0.01	0.98
9	0	0	Z	0	0	0	0.00	0.99
10	0	Z	Z	Z	0	0	0.00	0.99

Priors reverse jump for model selection: “rjhp = exp 0 1"								
Model	Transition rates						Frequency	Cumulative frequency
	q_{01}	q_{02}	q_{10}	q_{12}	q_{20}	q_{21}		
1	0	Z	0	0	Z	0	0.82	0.82
2	0	0	0	0	Z	0	0.10	0.92
3	0	0	0	Z	Z	0	0.02	0.94
4	0	0	Z	0	Z	0	0.02	0.95
5	0	Z	0	0	0	0	0.01	0.96
6	0	Z	Z	0	0	0	0.01	0.97
7	0	Z	0	Z	Z	0	0.01	0.97
8	0	0	Z	Z	Z	0	0.01	0.98
9	0	0	Z	0	0	0	0.00	0.98
10	0	Z	Z	Z	0	0	0.00	0.98

Table S6. Top 10 evolutionary models of primate social organization for the four-state scheme using 1000 different trees from the 10kTrees project (version 3;59). Independently from the hyperpriors used in model selection analyses, the top ten models account for >90% of the posterior sample. The most frequent model is the same across all the different hyperpriors employed. Z = zero. 0s and 1s indicate distinct non-zero transition rates. Subscripts on the rate coefficients correspond to the transitions between the four states of social organization (0: solitary; 1: pair living; 2: uni-male, 3: multi-male). Frequency = visits to the model in the posterior distribution of 75,000.

Priors reverse jump for model selection: "rjhp gamma 0 10 0 10"														
**Model**	**Transition rates**												**Frequency**	**Cumulative frequency**
	q_{01}	q_{02}	q_{03}	q_{10}	q_{12}	q_{13}	q_{20}	q_{21}	q_{23}	q_{30}	q_{31}	q_{32}		
1	0	Z	Z	0	Z	0	Z	Z	0	Z	0	0	0.71	0.71
2	0	Z	Z	0	Z	0	0	Z	0	Z	0	0	0.10	0.81
3	0	Z	Z	0	0	0	Z	Z	0	Z	0	0	0.05	0.86
4	0	Z	Z	0	Z	0	Z	Z	Z	Z	0	0	0.03	0.89
5	0	Z	Z	0	Z	0	0	0	0	Z	0	0	0.02	0.91
6	0	Z	Z	0	0	0	0	Z	0	Z	0	0	0.01	0.92
7	0	Z	Z	0	0	0	Z	0	0	Z	0	0	0.01	0.93
8	0	Z	Z	Z	0	0	0	Z	0	Z	0	0	0.01	0.94
9	0	Z	Z	0	Z	0	Z	0	Z	Z	0	0	0.01	0.95
10	0	Z	0	0	Z	0	Z	Z	0	Z	0	0	0.01	0.96

Priors reverse jump for model selection: "rj uniform -100 100"														
**Model**	**Transition rates**												**Frequency**	**Cumulative frequency**
	q_{01}	q_{02}	q_{03}	q_{10}	q_{12}	q_{13}	q_{20}	q_{21}	q_{23}	q_{30}	q_{31}	q_{32}		
1	0	Z	Z	0	Z	0	Z	Z	0	Z	0	0	0.72	0.72
2	0	Z	Z	0	Z	0	0	Z	0	Z	0	0	0.09	0.81
3	0	Z	Z	0	0	0	Z	Z	0	Z	0	0	0.05	0.86
4	0	Z	Z	0	Z	0	Z	Z	Z	Z	0	0	0.03	0.89
5	0	Z	Z	0	Z	0	0	0	0	Z	0	0	0.02	0.91
6	0	Z	Z	0	0	0	0	Z	0	Z	0	0	0.01	0.92
7	0	Z	Z	0	0	0	Z	0	0	Z	0	0	0.01	0.94
8	0	Z	Z	Z	0	0	0	Z	0	Z	0	0	0.01	0.95
9	0	Z	Z	0	Z	0	Z	0	Z	Z	0	0	0.01	0.96
10	0	Z	0	0	Z	0	Z	Z	0	Z	0	0	0.01	0.96

Priors reverse jump for model selection: "rjhp = exp 0 100"														
**Model**	**Transition rates**												**Frequency**	**Cumulative frequency**
	q_{01}	q_{02}	q_{03}	q_{10}	q_{12}	q_{13}	q_{20}	q_{21}	q_{23}	q_{30}	q_{31}	q_{32}		
1	0	Z	Z	0	Z	0	Z	Z	0	Z	0	0	0.72	0.72
2	0	Z	Z	0	Z	0	0	Z	0	Z	0	0	0.09	0.81
3	0	Z	Z	0	0	0	Z	Z	0	Z	0	0	0.05	0.86
4	0	Z	Z	0	Z	0	Z	Z	Z	Z	0	0	0.03	0.89
5	0	Z	Z	0	Z	0	0	0	0	Z	0	0	0.02	0.91
6	0	Z	Z	0	0	0	0	Z	0	Z	0	0	0.01	0.92
7	0	Z	Z	0	0	0	Z	0	0	Z	0	0	0.01	0.94
8	0	Z	Z	Z	0	0	0	Z	0	Z	0	0	0.01	0.95
9	0	Z	Z	0	Z	0	Z	0	Z	Z	0	0	0.01	0.96
10	0	Z	0	0	Z	0	Z	Z	0	Z	0	0	0.01	0.96

**Priors reverse jump for model selection: "rjhp exp 10"**														
**Model**	**Transition rates**												**Frequency**	**Cumulative frequency**
	q_{01}	q_{02}	q_{03}	q_{10}	q_{12}	q_{13}	q_{20}	q_{21}	q_{23}	q_{30}	q_{31}	q_{32}		
1	0	Z	Z	0	Z	0	Z	Z	0	Z	0	0	0.72	0.72
2	0	Z	Z	0	Z	0	0	Z	0	Z	0	0	0.09	0.82
3	0	Z	Z	0	0	0	Z	Z	0	Z	0	0	0.05	0.86
4	0	Z	Z	0	Z	0	Z	Z	Z	Z	0	0	0.03	0.89
5	0	Z	Z	0	Z	0	0	0	0	Z	0	0	0.02	0.91
6	0	Z	Z	0	0	0	0	Z	0	Z	0	0	0.01	0.92
7	0	Z	Z	0	0	0	Z	0	0	Z	0	0	0.01	0.94
8	0	Z	Z	Z	0	0	0	Z	0	Z	0	0	0.01	0.95
9	0	Z	Z	0	Z	0	Z	0	Z	Z	0	0	0.01	0.95
10	0	Z	0	0	Z	0	Z	Z	0	Z	0	0	0.01	0.96

**Priors reverse jump for model selection: "rjhp = exp 0 1"**														
**Model**	**Transition rates**												**Frequency**	**Cumulative frequency**
	q_{01}	q_{02}	q_{03}	q_{10}	q_{12}	q_{13}	q_{20}	q_{21}	q_{23}	q_{30}	q_{31}	q_{32}		
1	0	Z	Z	0	Z	0	Z	Z	0	Z	0	0	0.72	0.72
2	0	Z	Z	0	Z	0	0	Z	0	Z	0	0	0.09	0.81
3	0	Z	Z	0	0	0	Z	Z	0	Z	0	0	0.05	0.86
4	0	Z	Z	0	Z	0	Z	Z	Z	Z	0	0	0.03	0.89
5	0	Z	Z	0	Z	0	0	0	0	Z	0	0	0.02	0.91
6	0	Z	Z	0	0	0	0	Z	0	Z	0	0	0.01	0.92
7	0	Z	Z	0	0	0	Z	0	0	Z	0	0	0.01	0.93
8	0	Z	Z	Z	0	0	0	Z	0	Z	0	0	0.01	0.94
9	0	Z	Z	0	Z	0	Z	0	Z	Z	0	0	0.01	0.95
10	0	Z	0	0	Z	0	Z	Z	0	Z	0	0	0.01	0.96

Table S7. Average number of transitions inferred across 10,000 stochastic maps using SIMMAP function in R. (**a**) 3-state scheme and (**b**) 4-state scheme

a) 3-state scheme

	Solitary (S)	Pair living (P)	Group living (G)
**Solitary (S)**	-	9.375	0
**Pair living (P)**	4.472	-	7.147
**Group living (G)**	0	5.933	-

*10,000 trees with a mapped discrete character with 3 states (S, P, G). Trees have 26.927 changes between states on average.

b) 4-state scheme

	Solitary (S)	Pair living (P)	Uni-Male (UM)	Multi Male (MM)
**Solitary (S)**	-	10.036	0	0
**Pair living (P)**	3.803	-	0	8.599
**Uni-Male (UM)**	0	0	-	5.437
**Multi Male (MM)**	0	5.065	10.250	-

*10,000 trees with a mapped discrete character with 4 states (S, P, UM, MM). Trees have 43.192 changes between states on average.

Table S8. Proportion of pairs among primate social units with at least one pair. N: number of social units censused**.** Data were used for the analysis depicted in Figure 4.

**Species**	**% pairs**	**N**
Callithrix jacchus	3.8	52
Eulemur macaco	6.3	32
Eulemur coronatus	8.8	57
Saguinus mystax	9.0	100
Saguinus illigeri	11.8	17
Propithecus verreauxi	12.0	225
Saguinus wedelli	12.3	65
Saguinus nigrifrons	15.4	13
Nomascus concolor	17.6	17
Nomascus nasutus	17.6	17
Hapalemur alaotrenis	22.2	36
Pithecia aequatorialis	33.3	6
Eulemur mongoz	47.1	70
Saguinus imperator	50.0	2
Indri indri	55.6	18
Symphalangus syndactylus	60.0	10
Eulemur rubriventer	64.3	14
Lepilemur ruficaudatus	66.7	6
Hylobates lar	71.2	146
Tarsius sangirensis	75.0	4
Tarsius spectrum	80.8	26
Callicebus discolor	83.3	6
Hoolock hoolock	83.3	6
Lepilemur leucopus	83.3	12
Cheirogaleus medius	88.9	9
Nomascus gabriellae	88.9	18
Tarsius lariang	90.9	11
Aotus azarae	100.0	18
Avahi meridionalis	100.0	2
Avahi occidentalis	100.0	6
Hapalemur aureus	100.0	2
Hylobates agilis	100.0	10
Hylobates klossi	100.0	11
Hylobates klossi	100.0	16
Hylobates mulleri	100.0	2
Lepilemur edwardsi	100.0	6
Phaner pallescens	100.0	7
Tarsius dianae	100.0	6
